# Large-scale and high-resolution mass spectrometry-based proteomics profiling defines molecular subtypes of esophageal cancer for therapeutic targeting

**DOI:** 10.1038/s41467-021-25202-5

**Published:** 2021-08-16

**Authors:** Wei Liu, Lei Xie, Yao-Hui He, Zhi-Yong Wu, Lu-Xin Liu, Xue-Feng Bai, Dan-Xia Deng, Xiu-E Xu, Lian-Di Liao, Wan Lin, Jing-Hua Heng, Xin Xu, Liu Peng, Qing-Feng Huang, Cheng-Yu Li, Zhi-Da Zhang, Wei Wang, Guo-Rui Zhang, Xiang Gao, Shao-Hong Wang, Chun-Quan Li, Li-Yan Xu, Wen Liu, En-Min Li

**Affiliations:** 1 Guangdong Provincial Key Laboratory of Infectious Diseases and Molecular Immunopathology, the Key Laboratory of Molecular Biology for High Cancer Incidence Coastal Chaoshan Area, Shantou University Medical College, Shantou, Guangdong China; 2 College of Science, Heilongjiang Institute of Technology, Harbin, Heilongjiang China; 3 State Key Laboratory of Cellular Stress Biology, School of Pharmaceutical Sciences, Xiamen University, Xiamen, Fujian China; 4 Fujian Provincial Key Laboratory of Innovative Drug Target Research, School of Pharmaceutical Sciences, Xiamen University, Xiamen, Fujian China; 5 Shantou Central Hospital, Affiliated Shantou Hospital of Sun Yat-Sen University, Shantou, Guangdong China; 6 School of Medical Informatics, Daqing Campus, Harbin Medical University, Daqing, Heilongjiang China

**Keywords:** Drug development, Tumour biomarkers, Proteomics

## Abstract

Esophageal cancer (EC) is a type of aggressive cancer without clinically relevant molecular subtypes, hindering the development of effective strategies for treatment. To define molecular subtypes of EC, we perform mass spectrometry-based proteomic and phosphoproteomics profiling of EC tumors and adjacent non-tumor tissues, revealing a catalog of proteins and phosphosites that are dysregulated in ECs. The EC cohort is stratified into two molecular subtypes—S1 and S2—based on proteomic analysis, with the S2 subtype characterized by the upregulation of spliceosomal and ribosomal proteins, and being more aggressive. Moreover, we identify a subtype signature composed of ELOA and SCAF4, and construct a subtype diagnostic and prognostic model. Potential drugs are predicted for treating patients of S2 subtype, and three candidate drugs are validated to inhibit EC. Taken together, our proteomic analysis define molecular subtypes of EC, thus providing a potential therapeutic outlook for improving disease outcomes in patients with EC.

## Abstract (web summary)

Proteomics can aid in the identification of molecular subtypes in cancers. Here, the authors perform proteomic profiling of 124 paired oesophageal cancer and adjacent non-tumour tissues and identify two subtypes that are associated with patient survival for therapeutic targeting.

## Introduction

Esophageal cancer (EC) is one of the most aggressive cancer types and is the fourth leading cause of cancer-related deaths in China^1,2^. Although treatments have greatly improved in the past years, the 5-year overall survival (OS) rate of EC remains in the range of 15–25%; patients largely benefit from early diagnosis^3^. Several genomic analyses in EC have been performed to link genomic alterations with phenotypes, revealing several driver genes, such as *TP53*, *RB1*, *ZNF750*, *NOTCH1*, *FAT1*, and *NFE2L2*, with high frequencies of mutations^4–8^.Genomic alterations are largely translated into changes in protein levels, and protein functions are further modulated by post-translational modifications (PTMs). Both protein levels and PTMs are major determinants of cell phenotypes. Therefore, proteomic and phosphoproteomic analyses may provide additional insights into tumor biology that cannot be deciphered by genomic analysis. Large-scale, mass spectrometry (MS)-based proteomics have identified novel cancer subtypes and therapeutic targets for patients with colon, ovarian, breast, gastric, and liver cancers^9–20^, and provided valuable resources that have expanded our understanding of these cancers. However, the proteomic landscape of EC has not been characterized in a large cohort of patients. Furthermore, unlike breast and gastric cancer, no molecular subtypes based on proteomics study have been identified for EC that can assist patient stratification and therapeutic development^21–23^.

Here, we perform proteomic analysis of 124 paired EC tumor and the corresponding adjacent non-tumor tissues (cohort 1). Proteomic analyses reveal a catalog of proteins, phosphosites, and pathways that are dysregulated in ECs. Proteomic subtyping identify two subtypes that are associated with patient survival. Subtype diagnostic and prognostic models are constructed for clinical utilization. Furthermore, several potential drugs that specific for malignant subtype S2 are predicted and verified by functional experiments, which might provide new therapeutic opportunities to improve treatment outcome of EC.

## Results

### Large-scale proteomic and phosphoproteomic analysis of esophageal cancer (EC)

**Fig. 1:**
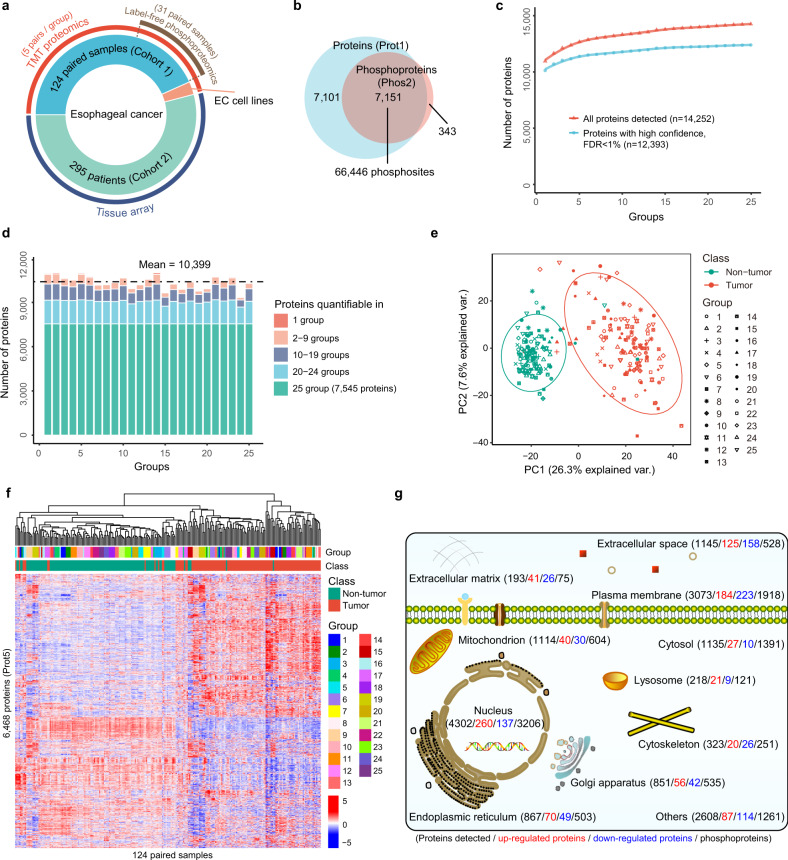
Large-scale proteomic and phosphoproteomic analysis of esophageal cancer (EC). **a** Summary of EC samples and cell lines for proteomic, phosphoproteomic and/or immunohistochemical analysis. One hundred and twenty-four paired EC tumor and adjacent normal samples (cohort 1) were divided into 25 groups for TMT proteomics, and 31 paired samples were subjected to lable-free phosphoproteomics. EC tumor samples from 295 patients (cohort 2) were used for immunohistochemistry. **b** The overlap of proteins and phosphoproteins. Seven thousand one hundred and fifty-one proteins were identified with 66,446 phosphosites. Seven thousand one hundred and one proteins were identified with only their non-phosphorylated forms. Three hundred and forty-three proteins were identified with only their phosphorylated forms. Prot1: proteins with quantified values in at least one of those 25 groups of samples used for proteomic analysis as shown in **a**; Phos2: phosphorylation sites with quantified values in at least one of those 31 pairs of samples used for phosphoproteomics analysis as shown in **a**. **c** Cumulative number of proteins quantified in 25 groups of samples. **d** Distribution of the number of groups in which the proteins were quantifiable. Ten thousand six hundred and ninety-three proteins were identified in ≥10 groups, and 7545 proteins were quantified in all 25 groups. **e** Principle component (PC) analysis of the TMT proteomic data separated tumor samples from non-tumor samples, and no batch effects were observed. Samples analyzed in different TMT groups (batches) are shown with different shapes. Tumor and non-tumor samples are colored in red and green, respectively. The ellipse presents the 0.9 confidence intervals for each type. Var.: variation. **f** Hierarchical clustering of the 124 paired tumor and non-tumor samples. **g** Subcellular distribution of all proteins, upregulated proteins, downregulated proteins, and phosphoproteins detected.

We performed isobaric tandem mass tag (TMT)-based proteomic analysis for 124 pairs of EC tumor and the corresponding adjacent non-tumor tissues (cohort 1), which identified 14,252 proteins in total, with 10,399 proteins per group on average (five pairs/group) (Fig. 1a–d and Supplementary Fig. 1a–b and Supplementary Data 1a). Of these 14,252 proteins, 9300 and 6468 proteins were quantifiable in at least half of the samples and all samples, respectively (Supplementary Fig. 2a, b and Supplementary Data 1b). The protein expression ratios showed a globally homogeneous distribution among the groups. No clear quantitative shifts were observed, especially so for proteins quantifiable in all samples (Prot5), which had fewer outliers (Supplementary Fig. 2c, d). Principal component analysis and hierarchical clustering revealed that tumors could be well separated from non-tumors. Additionally, no batch effects were observed among groups, supporting the high quality of our proteomic data (Fig. 1e, f). As protein functions are often modulated by PTMs, especially phosphorylation, we performed label-free phosphoproteomics for 31 pairs of tumor and non-tumor esophageal tissues. The phosphoproteomics procedure was highly reproducible (Supplementary Fig. 2e, f). In total, we identified 73,651 phosphosites in 7943 proteins (Fig. 1a and Supplementary Figs. 1b, 2g and Supplementary Data 1c), of which 67,393 phosphosites in 7494 proteins were quantifiable (Fig. 1b). The cumulative number of phosphosites (phosphoproteins) increased steadily and approached a plateau when the sample size reached approximately 36 (18 pairs), suggesting that 31 paired samples were sufficient to capture the phosphoproteomic landscape in EC (Supplementary Fig. 2h, i). A case-by-case inspection revealed that although the number of phosphosites identified in each patient did not differ much, like the phosphoproteins, tumor samples in general had a higher number of phosphosites than non-tumor samples (Supplementary Fig. 2j, k). We then analyzed the subcellular distribution of the quantified proteins and phosphoproteins, revealing that most of them were mainly distributed in the nucleus, plasma membrane, cytosol, mitochondria, and extracellular space, which was consistent with previous reports (Fig. 1g and Supplementary Data 1d)^15^.

### Dysregulated proteins and pathways were identified by proteomic analysis

**Fig. 2:**
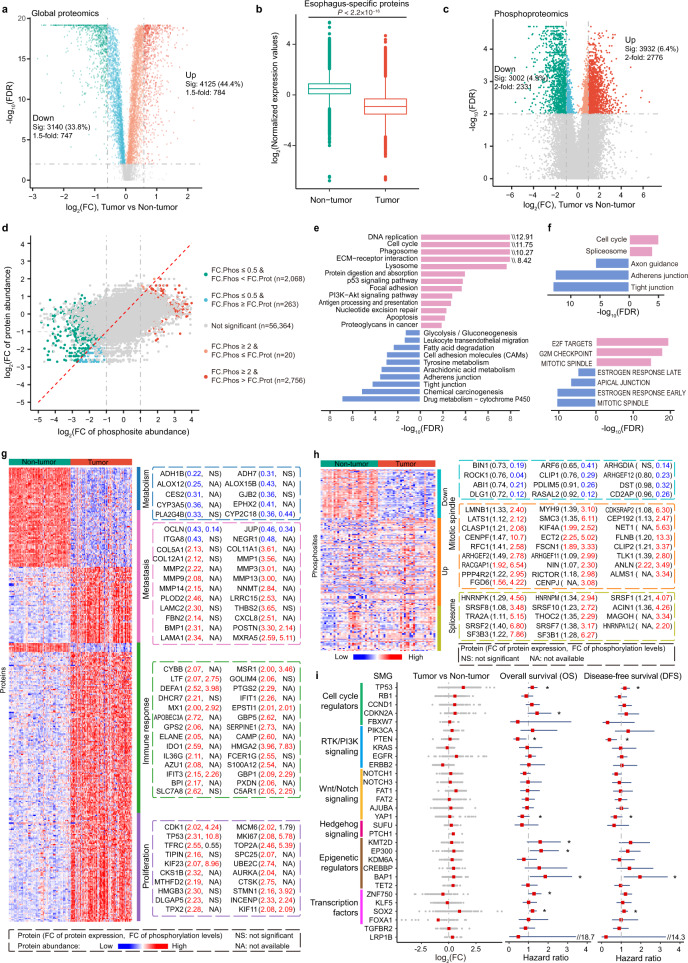
Dysregulated proteins and pathways were identified by proteomic analysis. **a** Volcano plot indicating proteins upregulated or downregulated in tumors. Light red and green colors represent proteins with BH adjusted *P* value (< 0.01) (Sig), whereas red and green represent proteins with BH adjusted *P* value (< 0.01) and more than 1.5-fold change. Other proteins are colored in gray. *P* values were calculated using the two-sided Wilcoxon signed-rank test. **b** Box plot of log_2_-transformed fold change of esophageal-specific proteins (Tumor, *n* = 124; Non-tumor, *n* = 124). *P* value was calculated using the two-sided Wilcoxon rank-sum test. In the box plots, the middle bar represents the median, and the box represents the interquartile range; bars extend to 1.5× the interquartile range. **c** Volcano plot indicating phosphosites upregulated and downregulated in tumors. Colors are the same as describe in **a** except that red and green represent proteins with more than 2-fold change. **d** Comparison of the changes of phosphosite abundance (FC.Phos) with those of the corresponding protein abundance (FC.Prot). The red dashed line indicates the diagonal line. Green and light green colors indicate significantly downregulated phosphosites (BH adjusted *P* value < 0.01 and FC.Phos ≤ 0.5), whereas green further requires FC.Phos < FC.Prot. Red and light red colors indicate significantly upregulated phosphosites (BH adjusted *P* value < 0.01 and FC.Phos ≥ 2), whereas red further requires FC.Phos > FC.Prot. Other phosphosites are colored in gray. *P* values were calculated using the two-sided Wilcoxon signed-rank test. **e** Enriched KEGG pathways for differential proteins colored by red and green as shown in **a**. Pink bars indicate pathways enriched in the upregulated proteins (*n* = 784). Blue bars indicate pathways enriched in the downregulated proteins (*n* = 747). **f** KEGG pathways (top) and hallmark get sets (bottom) enriched for differential phosphoproteins. Pink bars indicate pathways enriched in the upregulated phosphoproteins (*n* = 1040). Blue bars indicate pathways enriched in the down-regulated phosphoproteins (*n* = 576). **g** Heat map representation of the expression levels of selected, differential expressed proteins between tumor and non-tumor samples (FC >1.5 or <0.67). Functional categories related to selected proteins are denoted beside the heat map. The right panel shows the proteins whose expression levels changed larger than 2-fold between tumor and non-tumor samples, and that are significant correlated with patient risk. The two-sided log-rank *P* values (without correction for multiple testing) were calculated by the Xtile method. **h** Heat map representation of the phosphorylation levels of differential phosphosites. The right panel shows the phosphoproteins that changed larger than 2-fold in phosphorylation abundance between tumor and non-tumor samples. **i** Protein expression variations of significantly mutated genes (SMGs). Left, log_2_-transformed fold change between paired tumor (*n* = 124) and non-tumors (*n* = 124) (mean in red). Middle, the red points indicate the overall survival hazard ratios of SMGs, and the endpoints represent lower or upper of the 95% confidence intervals. Right, the red points indicate the disease-free survival hazard ratios, and the endpoints represent lower or upper of the 95% confidence intervals. *Cox *P* value < 0.1. The two-sided Cox *P* values (without correction for multiple testing) were calculated using the Cox PH model.

We next sought to identify the proteins that were differentially expressed between tumor and non-tumor samples. Among the 9300 proteins that were quantifiable, 4125 (44.4%) and 3,140 (33.8%) were significantly upregulated and downregulated, respectively, in tumors compared to paired non-tumors (Benjamini-Hochberg (BH) adjusted *P* < 0.01, Wilcoxon signed-rank test). Of these, 1531 proteins exhibited a fold-change larger than 1.5, with 784 and 747 being up-regulated and downregulated in tumors, respectively (Fig. 2a and Supplementary Data 2a). Interestingly, the proteins that were altered in tumors were exceptionally enriched in proteins that were localized in the extracellular matrix (34.7%) and extracellular space (24.7%) (Fig. 1g and Supplementary Data 1d), suggesting that the tumor microenvironment had undergone drastic changes during tumorigenesis. In addition, we found that most of the esophagus-specific proteins annotated in the Human Protein Atlas such as KRT4, KLK13, KRT78, SPINK5, SPINK7, and CRNN (Supplementary Fig. 3a), were significantly downregulated in tumors (Fig. 2b and Supplementary Data 2b), indicating loss of esophagus identity as a characteristic of EC. We also assessed the differences in the abundance of phosphosites between the 31 pairs of tumor and adjacent non-tumor samples. Among the 61,471 phosphorylation sites quantifiable in at least half of the samples, 3932 (6.4%) and 3,002 (4.9%) were significantly upregulated and downregulated in tumors compared to paired non-tumors, respectively (BH adjusted *P* < 0.01, Wilcoxon signed-rank test). Of these, 5107 phosphosites exhibited a fold-change larger than 2, with 2776 and 2331 being upregulated and downregulated in tumors, respectively (Fig. 2c and Supplementary Data 2c). The fold-change of phosphorylation on the vast majority of phosphosites was greater than that of the corresponding protein levels (Fig. 2d).

Functional enrichment analysis of differentially expressed proteins revealed that cell cycle, DNA repair, immune response, and epidermal mesenchymal transition (EMT) pathways, among others, were over-represented in proteins that were upregulated in tumors. Whereas, metabolism and estrogen response-related pathways were over-represented in the proteins that were downregulated (Fig. 2e and Supplementary Fig. 3b and Supplementary Data 2d). The downregulation of estrogen response-related proteins was consistent with previous studies^24,25^, suggesting a potential link between estrogen signaling and EC. In addition to proteins involved in the cell cycle, EMT, and estrogen response-related pathways, the phosphoproteomic data revealed that proteins in the spliceosome were often highly phosphorylated in tumors (Fig. 2f and Supplementary Data 2e). The representative differentially expressed proteins with implications in proliferation, immune response, metastasis, and metabolism as well as hyperphosphorylated proteins with implications in spliceosome are shown (Fig. 2g, h and Supplementary Data 2f, g). A number of interferon-stimulated genes (ISGs) were found to be highly induced in tumor compared to non-tumor samples, such as GBP1, CAMP, PTGS2, GOLIM4, IFIT3, MX1, LTF, and CYBB (Fig. 2g). The expression of representative ISGs including MX1, OAS3, and IFIT1 was found to be significantly higher in tumor cells compared to stroma cells (Supplementary Fig. 3c). Interestingly, for many proteins such as CDK1, TP53, STMN1, MKI67, TOP2A, and KIF23, not only their expression levels, but also the rates of phosphorylation were significantly upregulated in tumors. Spliceosomal proteins including SRSF1, SRSF2, SRSF7, SRSF8, and SRSF10 were found to be hyperphosphorylated; the phosphorylation of these proteins often occurred on serine residues such as S199, S202, S234, and S238 of SRSF1, and S206, S208, S212, and S220 of SRSF2, in the RS domain (Supplementary Data 2c). Phosphorylation in this functional domain is well known to be important in regulating the assembly and disassembly of spliceosomes, and alternative splicing in genes^26,27^. Our data strengthened the notion that phosphoproteomics could complement proteomics in understanding the biology of EC.

We then performed PTM-SEA analysis for differential phosphosites between tumor and non-tumor samples. Our results revealed that 16 signatures were significantly upregulated, while 65 were downregulated in tumor samples (paired two-sided Student’s *t*-test, BH adjusted *P* < 0.01) (Supplementary Fig. 3d, e). Among these significantly changed signatures, 45, 21, and 15 were perturbation signatures, kinase-substrate signatures, and signatures of molecular signaling pathway, respectively (Supplementary Fig. 3d, e). For instance, kinase-substrate signatures including CDK1, CDK2, CDC7, CHEK2, MELK, CDK7, IKK, PIM1, and CK2A1 were upregulated, while RET, PKCZ, FYN, AXL, SGK1, MKK4, p90RSK, MEK1, ABL1, LCK, EGFR, and EPHA2 were downregulated in tumor compared to non-tumor samples (Supplementary Fig. 3e).

We next compared our proteomics and phosphoproteomics data to previously published genomic data. Genomic analysis has identified a large number of frequently mutated genes and pathways in EC^4,5,7,28–32^. Of the 35 significantly mutated genes (SMGs) we collected from the literature^4,5,7,28^, 30 of them were quantifiable in our proteomics data (Fig. 2i and Supplementary Data 3a). Seventeen SMGs showed a fold-change greater than 1.2 in protein expression between tumors and non-tumors. In particular, TP53 was upregulated, whereas ERBB2 and ZNF750 were downregulated in tumors (fold-change >1.5) (Supplementary Data 2a). Downregulation of ZNF750 was consistent with previous studies showing that ZNF750 is expressed at a very low levels via truncating mutations and functions as a tumor suppressor in EC^5,7^. Of those 30 SMGs quantifiable in our proteomics data, nine were associated with survival outcomes. Specifically, cell cycle regulators, TP53 and CDKN2A; epigenetic regulators, KMT2D, EP300, and BAP1; and transcription factors, ZNF750 and SOX2, were positively correlated, while transcription factors, PTEN and YAP1, were negatively correlated with survival risk.

Signaling pathways including the cell cycle, RTK-PI3K, Wnt, and Notch pathways are frequently mutated in EC^4,7^. Based on our proteomic data, a large cohort of cell cycle-related proteins such as *CCND1* and *CDK6*; or *TP53*, *RB1*, *CDKN2A*, *CHEK1*, and *CHEK2*, regardless of whether they were amplified or mutated at the genomic level, were upregulated (Supplementary Fig. 3f, g and Supplementary Data 3b). With the integration of our phosphoproteomic data, we found numerous functional phosphosites in these proteins to be hyperphosphorylated in tumors. For instance, the phosphorylation of TP53 at S315, which could enhance TP53 transactivation potential through nuclear retention and promote MDM2-dependent proteolysis of TP53^33,34^, was increased dramatically in tumors (fold-change >10) (Supplementary Data 3c). In addition, RB1, a tumor suppressor which prevents cell proliferation by inhibiting E2F transcriptional activities, was negatively regulated by its phosphorylation^35^. Phosphorylation of T373 and S795 on RB1 increased significantly in tumors (Supplementary Data 3c), suggesting that RB1 phosphorylation might counteract its overexpression^12^. Despite proteins in the RTK / PI3K pathway exhibiting minor changes at the protein level, the phosphorylation levels were significantly altered in the tumors (Supplementary Fig. 3f). The upregulation of EGFR protein expression was consistent with its amplification in ECs. Although mutations and amplifications for proteins downstream of the EGFR signaling cascade, including KRAS, MRAS, RAF1, AKT1, SOS1, SOS2, and PIK3CA, occur in 50.6% of cases in EC, no significant changes in protein expression were observed^4^. Genomically altered genes in the Wnt pathway such as CTNNB1, SFRP4, and DAAM2, were also altered at the protein level. Although genomic alterations have been reported for genes in Notch signaling such as NOTCH1, NOTCH2, and NOTCH3, no significant changes were observed (Supplementary Fig. 3f, g).

We also compared our proteomics and phosphoproteomics data for those variable metastasis and immune response-related genes shown in Fig. 2g to previously published genomic data. It should be noted that only the percentage of SNV/Indel, but not that of amplification or deletion, is available for these genes. No genomic alterations were found for many of the metastasis and immune response genes such as MMP1, MMP2, MMP3, MMP9, MX1, IDO1, IFIT1, and IFIT3 (Supplementary Fig. 3h, i). The amplification of genes such as COL5A1, IDO1, and GOLIM4 were in accordance with their higher expression in tumor samples (Supplementary Fig. 3h, i), whereas no significant change of protein or phosphorylation levels was observed for genes with genomic amplification, such as CD4, CD7, LIME1, UNC93B1, C1S, C1R, or C8G. Similarly, frequently mutated genes such as BTN2A1 (3.4%), RHOA (2.3%), and EPHB4 (2.3%) exhibited no changes at protein level^4^. This further highlights the necessity of performing proteomic study to uncover targets for cancer therapy that are undetectable by genomic sequencing. In addition, it is worth-noting that the protein expression and phosphorylation levels of a large cohort of epigenetic regulators such as histone methyltransferase and demethylases KDM1A, KDM3A, KDM3B, KDM5C, ASH1L, NSD2, and NSD3; histone acetyltransferase EP300, CREBBP, and KMT2D; and chromatin structure modifier ARID1A, SMARCC1, and SMARCC2, were upregulated in tumors, and their expression levels were positively correlated with survival risk^4,5,7,28–32^ (Fig. 2i and Supplementary Fig. 3f). However, we also observed that the increased expression levels of some proteins which have been reported to harbor frequent inactivating mutations such as EP300, CREBBP, BAP1, KMT2D, KMT2C, and KMT6A, were inconsistent with their genomic alterations^7,30,36,37^. Collectively, the connections and discordances between proteomic and genomic alterations provided valuable clues to decipher the pathogenesis of EC.

### Proteomic analysis stratified patients into two subtypes, the low-risk S1 subtype and the high-risk S2 subtype

**Fig. 3:**
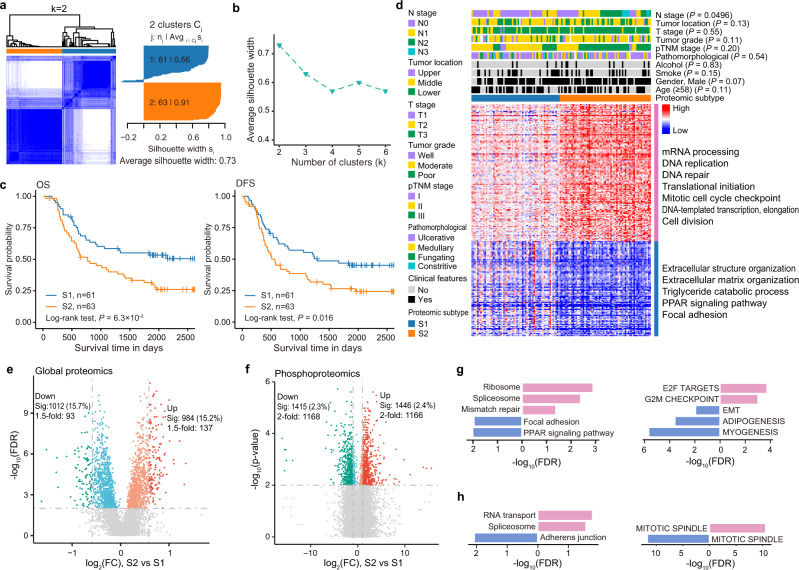
Molecular subtypes of EC were defined by proteomic analysis. **a** Consensus clustering of EC tumor samples. The left panel shows consensus matrices of the 124 EC samples with two clusters (*k* = 2). Consensus clustering was performed on the top 25% most-variant proteins in Prot5 as described in Supplementary Fig. 2a. The right panel shows the silhouette-width plot. **b** Average silhouette-width plot. The average silhouette width takes the maximum value when number of clusters was 2 (*k* = 2). **c** Kaplan–Meier curves of overall survival (left) and disease-free survival (right) for subtype S1 and S2. *P* values were calculated by two-sided log-rank test. **d** Heatmap representation of the relative protein abundance of differentially expressed proteins between S2 and S1 (BH adjusted *P* value < 0.01, FC > 1.5 or <0.67). The upper panel shows the association between molecular subtypes and clinicopathologic characteristics. GO (gene ontology) biological functions related to these proteins are denoted on the right. The *P* values were calculated by chi-squared test. **e** Volcano plot indicating proteins upregulated and downregulated in subtype S2. Light red and green colors represent proteins with BH adjusted *P* value < 0.01 (Sig), whereas red and green represent proteins with BH adjusted *P* value < 0.01 and fold change more than 1.5. Other proteins are colored in gray. *P* values were calculated using the two-sided Wilcoxon rank-sum test. **f** Volcano plot indicating phosphosites upregulated and downregulated in subtype S2. Colors are the same as in **e** except that red and green represent proteins with fold change more than 2. *P* values were calculated using the two-sided Wilcoxon rank-sum test. **g** KEGG pathways (left) and hallmark get sets (right) enriched in differentially expressed proteins between subtype S1 and S2. Pink bars indicate pathways enriched in the upregulated proteins (*n* = 137). Blue bars indicate pathways enriched in the downregulated proteins (*n* = 93). **h** KEGG pathways (left) and hallmark get sets (right) enriched for phosphoproteins with differential phosphorylation between subtype S1 and S2. Pink bars indicate pathways enriched in the upregulated phosphoproteins (*n* = 541). Blue bars indicate pathways enriched in the downregulated phosphoproteins (*n* = 519).

We next sought to investigate if the tumor samples could be stratified into clinically relevant molecular subtypes based on our proteomic analysis. Principal component analysis (Fig. 1e) and hierarchical clustering (Fig. 1f) of our proteomics data demonstrated a clear distinction between tumor and non-tumor tissues, which further highlighted the heterogeneity among tumor samples that underpins our subtyping analysis. We employed consensus clustering for the top 25% of the most variable proteins, and identified two major subtypes that had maximal average silhouette width, which we named as S1 and S2 subtypes (Fig. 3a, b and Supplementary Fig. 4a). The S1 and S2 subtypes contained 61 and 63 tumor samples, respectively (Supplementary Data 4a). Patients with the S2 subtype had worse OS and disease-free survival (DFS) outcomes compared to those in the S1 subtype (Fig. 3c). Specifically, the OS median was 1841 and 655 days for S1 and S2 subtypes, respectively (log-rank *P* = 6.3 × 10^-3^), and the DFS median was 1244 and 510 days for S1 and S2 subtypes, respectively (log-rank *P* = 0.016) (Fig. 3c). Cox regression analysis revealed that the subtypes we defined were significantly correlated for both OS (Hazard ratio = 1.899, Cox *P* = 7.25 × 10^−3^) and DFS (Hazard ratio = 1.726, Cox *P* value = 0.017), and could be used as an independent prognostic factor for both OS (*P* = 4.25 × 10^−3^) and DFS (*P* = 7.81 × 10^−3^) (Supplementary Data 4b). In addition, except for the N stage *(P* = 0.0496), there were no significant differences between the clinicopathological characteristics of patients in the S1 and S2 subtypes (Fig. 3d and Supplementary Data 4c).

To further characterize the two subtypes, we performed differential expression analysis for the 6468 proteins that were quantifiable in the 124 paired samples with high confidence (Supplementary Fig. 2a). A total of 984 and 1012 proteins were significantly upregulated and downregulated, respectively, in the S2 subtype compared to S1 subtype (BH adjusted *P* < 0.01, Wilcoxon rank-sum test). Of these, the expression levels of 230 proteins were altered by more than 1.5-fold, with 137 and 93 being upregulated and downregulated in the S2 subtype, respectively (Fig. 3e and Supplementary Data 4d). Of the 31 EC patients chosen for label-free phosphoproteomics, 15 patients belonged to S1 and 16 belonged to the S2 subtype (Supplementary Data 4a). Among the 61,471 phosphorylation sites quantifiable in at least half of the samples (Supplementary Fig. 2g), 1446 and 1415 sites were significantly increased and decreased, respectively, in the S2 samples (*P* < 0.01, Wilcoxon rank-sum test). As the vast majority of these significantly altered phosphosites exhibited fold changes larger than 2, it was not due to the change in the corresponding protein levels (Fig. 3f and Supplementary Fig. 4b and Supplementary Data 4e).

A heat map analysis of differentially expressed protein expression between S1 and S2 (Fig. 3d), revealed that the S2 subtype exhibited an extreme expression pattern; the upregulated proteins were expressed at an extremely high level, while downregulated proteins were expressed at an extremely low level. Indeed, there was a significant overlap between the differentially expressed proteins found in tumor versus non-tumor samples and those in S2 versus S1 subtypes (*P* = 2.02 × 10^−13^ and < 2.2 × 10^−16^ for upregulated and downregulated proteins, respectively; Supplementary Fig. 4c). The upregulated proteins in S2 were enriched in pathways such as mRNA processing, DNA replication, DNA repair, E2F targets, and G2/M checkpoint (Fig. 3d, g and Supplementary Fig. 4d and Supplementary Data 4f), which were also enriched in proteins upregulated in tumor samples when compared to non-tumor samples. This indicated that the activity of these pathways was progressively enhanced from non-tumor to S1 subtype-tumor to S2 subtype-tumor, which might lead to the malignant phenotype in subtype S2. The myogenesis-related pathway enriched in downregulated proteins also showed a similar trend (Fig. 3d, h and Supplementary Fig. 4d). Strikingly, focal adhesion and EMT-related pathways were found to be enriched with upregulated proteins in tumor samples versus non-tumor samples. However, in the S2 versus S1 subtype, they were enriched in downregulated proteins (Figs. 2e and 3g). The downregulation of many of these proteins such as ITGA7^38,39^, TIMP3^40,41^, ABI3BP^42^, MYLK, and MYL9^43^ (Supplementary Fig. 4d), have been reported to be associated with proliferation, invasion, and migration in various types of cancers—suggesting that this might serve as a potential mechanism underlying the malignant S2 subtype.

For proteins with differential phosphorylation in S2 versus S1 subtype, they were enriched in spliceosomes, adherens junctions, and mitotic spindle-related pathways, which were similar to those observed for proteins with differential phosphorylation in tumor versus non-tumor samples (Fig. 3h and Supplementary Data 4g). This indicated that aberrant phosphorylation of these pathways might play an important role in both the initiation and development of EC.

We then performed PTM-SEA analysis for differential phosphosites between the two subtypes. Our results revealed that 13 signatures were significantly upregulated, while 14 were downregulated in S2 subtype (unpaired two-sided Student’s *t*-test, *P* < 0.05) (Supplementary Fig. 4e, f). Among these significantly changed signatures, 10 and 17 are perturbation signatures and kinase-substrate signatures, respectively. No signatures of molecular signaling pathway were found to be significantly different between S1 and S2 subtype (Supplementary Fig. 4f). For instance, kinase-substrate signatures including AMPKA2, PKCI, MST2, PDK1, DYRK2, CDC7, and DYRK1A were upregulated, while CK1A, DNAPK, GSK3A, p70S6K, LYN, IKKA, CK1D, GSK3B, ATR, and p38A/MAPK14 were downregulated in S2 subtype compared to S1 subtype (Supplementary Fig. 4f).

### Subtype diagnostic signature composed of ELOA and SCAF4 were identified in EC

**Fig. 4:**
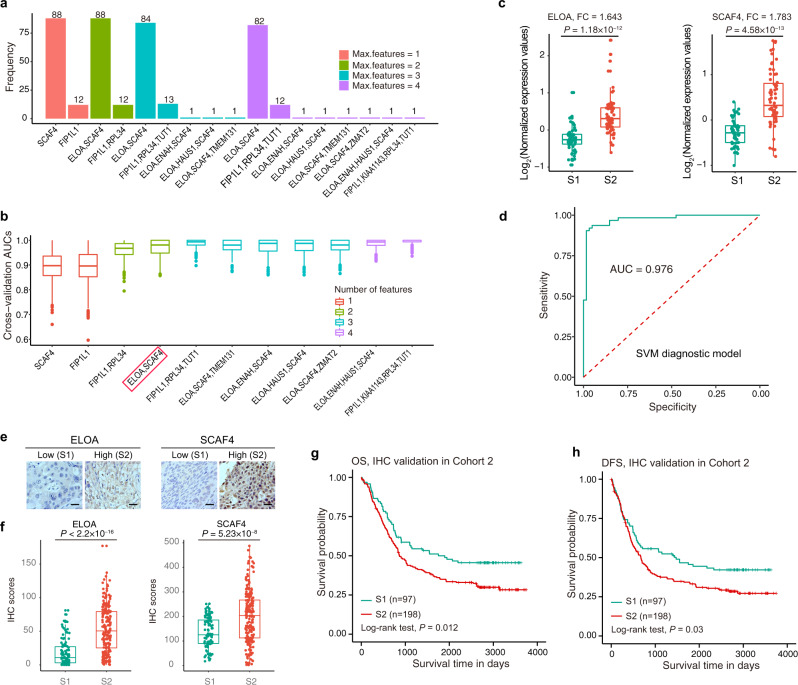
Subtype diagnostic signature composed of ELOA and SCAF4 were identified in EC. **a** Bar plots of the frequency of the signatures in 100 times feature selections. Red, green, cyan, and purple indicate that the maximum number of features is 1, 2, 3, and 4, respectively. **b** Box plots of the cross-validation AUCs (area under the ROC curve, *n* = 100) of the 11 signatures shown. **c** Box plots of log_2_-transformed protein expression ratios of ELOA (left) and SCAF4 (right) (S1, *n* = 61; S2, *n* = 63). *P* values are calculated by the two-sided Wilcoxon rank-sum test. **d** ROC curve of the SVM model with signature 4 (ELOA, SCAF4). **e** Representative IHC (immunohistochemistry) images of ELOA and SCAF4 protein expression in the independent EC Cohort (Cohort 2, *n* = 295). Scale bars, 100 µm. **f** Box plots of IHC scores of ELOA (left) and SCAF4 (right) in the predicted S1 and S2 patients in Cohort 2 (S1, *n* = 97; S2, *n* = 198). *P* values are calculated by the two-sided Wilcoxon rank-sum test. **g**, **h** Kaplan–Meier curves of OS (**g**) and DFS (**h**) for each predicted subtype in the independent EC Cohort 2. *P* values are calculated by two-sided log-rank test. In the box plots **b**, **c**, **f**), the middle bar represents the median, and the box represents the interquartile range; bars extend to 1.5× the interquartile range.

We next sought to identify subtype diagnostic signatures which might be usable in a clinical setting. To this end, we performed feature selection with the maximum number of features set to 1, 2, 3, or 4. Using the maximum area under the ROC curve (AUC) as the criterion, 11 unique signatures were identified, among which signature “ELOA, SCAF4” had the highest frequency (Fig. 4a and Supplementary Data 5a). Five hundred cross-validation experiments showed that signature “ELOA, SCAF4” yielded a favorable predictive performance (mean AUC = 0.970) (Fig. 4b and Supplementary Data 5b). ELOA, also known as elongin A, is a component of the SIII complex, which activates RNA polymerase II elongation by suppressing transient pausing of the polymerase at many sites within transcription units^44^. SCAF4, also known as splicing factor, arginine/serine-rich 15, belongs to the splicing factor SR family. It may act to physically and functionally link transcription and pre-mRNA processing^45,46^. Taking the number of features, stability (frequency), and prediction performance into consideration, we selected “ELOA, SCAF4” as the subtype diagnostic signature for subsequent analysis. Both ELOA and SCAF4 exhibited significantly higher expressions in the S2 subtype than in S1 (*P* = 1.18 × 10^−12^ and 4.58 × 10^−13^ for ELOA and SCAF4, respectively; Wilcoxon rank-sum test) (Fig. 4c). An SVM classification model with ELOA and SCAF4 as features was constructed for subtype prediction (http://www.licpathway.net/ECSubtype/analysis_diagnostic.html), and an AUC of 0.976 was obtained for the 124 patients we performed proteomics analysis on (Cohort 1) (Fig. 4d).

To further validate the subtype diagnostic model, we quantified the nuclear expression of ELOA and SCAF4 in an independent cohort of EC patients (*n* = 295, Cohort 2) by immunohistochemistry (IHC) (Supplementary Data 5c). Via this model, 97 patients were predicted to be of the S1 subtype and 198 patients of the S2 subtype (Supplementary Data 5d). IHC revealed that ELOA and SCAF4 exhibited stronger staining in S2 compared to S1 subtype (*P* < 2.2 × 10^−16^ and *P* = 5.23 × 10^−8^ for ELOA and SCAF4, respectively; Wilcoxon rank-sum test) (Fig. 4e, f). The subcellular localization of these two proteins remains unchanged between the two subtypes, with both predominantly localized in the nucleus of cells (Fig. 4e). Furthermore, Kaplan–Meier curves showed that patients with the S2 subtype had worse OS (OS median = 1414 and 855.5 days for S1 and S2, respectively; log-rank *P* = 0.012) (Fig. 4g) and DFS outcomes (DFS median = 1150 and 639.5 days for S1 and S2, respectively; log-rank *P* = 0.03) (Fig. 4h) than those in the S1 subtype, indicating that the subtype diagnostic model we constructed could accurately predict EC subtypes.

As the defined subtypes are based on proteomic data that are independent of the pTNM stage, we further investigated if the integration of the pTNM-defined subtypes can better stratify EC patients. We first constructed a prognostic model using Cox proportional hazards (Cox PH) model, in which the subtype and pTNM stage served as independent variables and survival information as dependent variable (referred to as “pTNM+Subtype” model). For patient stratification, we further constructed a “pTNM+Subtype 3c” model via k-means clustering (*k* = 3) on the risk scores predicted by “pTNM+Subtype” model (see “Methods” section for details on the two models; http://www.licpathway.net/ECSubtype/analysis_prognostic.html). At any time frame, ranging from one to seven years, both “pTNM+Subtype” and “pTNM+Subtype 3c” models showed larger time-dependent AUCs^47^ compared to the pTNM stage alone for both OS and DFS prediction (Supplementary Fig. 5a, b). ROC curves of the pTNM stage, “pTNM+Subtype”, and “pTNM+Subtype 3c” model for OS and DFS at five years were shown in Supplementary Fig. 5c, d. Furthermore, compared with the pTNM stage, “pTNM+Subtype 3c” model yielded a more significant (log-rank *P* value: 2.13 × 10^−4^ < 4.70 × 10^−3^ for OS and 1.20 × 10^−4^ < 8.80 × 10^−4^ for DFS) and reasonable stratification, allowing more patients being grouped under the low risk classification to avoid unnecessary treatment (Supplementary Fig. 5e–h). For those 295 patients in Cohort 2, although pTNM stage alone had provided an excellent stratification (Log-rank *P* = 1.34 × 10^−6^ for OS and 1.66 × 10^−6^ for DFS), the “pTNM+Subtype 3c” model exhibited better stratification for OS (log-rank *P* value: 7.92 × 10^−7^ < 1.34 × 10^−6^; low risk n: 79 vs. 24) (Supplementary Figs. 5i–l), suggesting “pTNM+Subtype 3c” model to be of potential clinical value in improving the pTNM staging system.

### Drug prediction and validation for EC based on molecular subtype defined

**Fig. 5:**
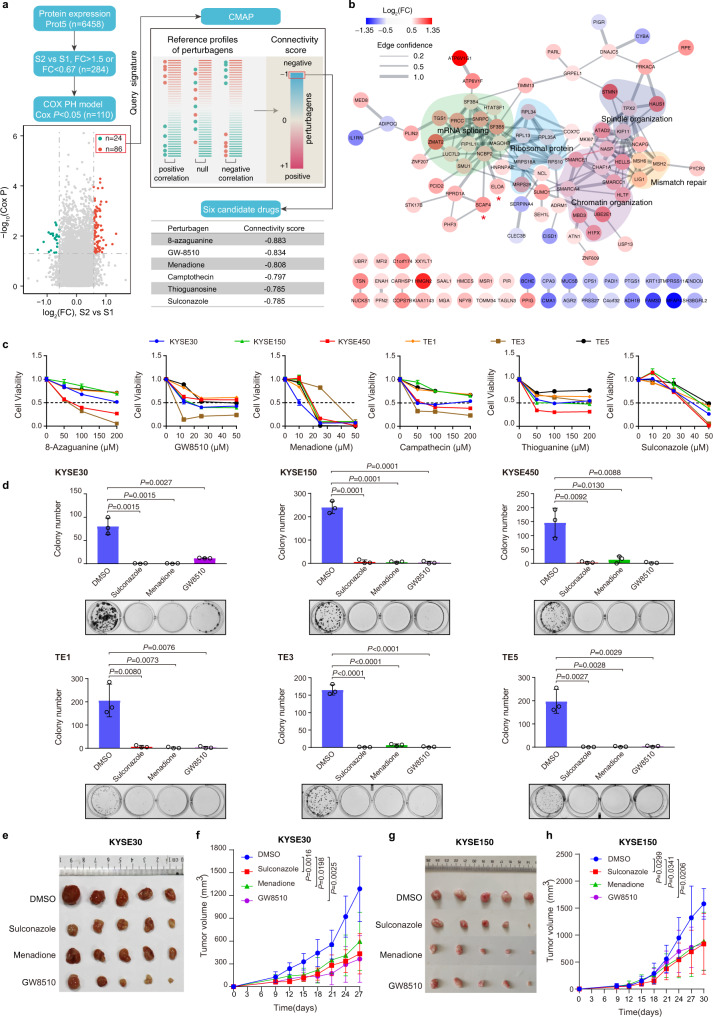
Drug prediction and validation for EC based on molecular subtype defined. **a** Workflow of drug prediction. Volcano plot indicates proteins that are differentially expressed between S1 and S2 and significantly associated with overall survival (Cox *P* value < 0.05). The two-sided Cox *P* values (without correction for multiple testing) were calculated using the Cox PH model. Red and green represent proteins with fold change larger than 1.5 between S1 and S2. Other genes are colored in gray. The 86 upregulated and 24 downregulated proteins were used as the query signature to match the reference profiles of perturbagens in CMAP to calculate connectivity scores. Perturbagens are sorted by connectivity score in increasing order, and the top perturbagens are predicted as candidate drugs. **b** Protein–protein interaction network of the query signature in **a**. The protein–protein interactions were obtained from the STRING database. The width of the line indicates the edge confidence. Upregulated proteins are colored in red, and downregulated proteins in blue. Several significantly enriched biological processes are highlighted by different colors. **c** Viabilities of six EC cell lines treated with six candidate drugs at concentrations as indicated for 24 h. Representative data from four biological repeats was shown (mean ± SD). **d** Colony formation assays of six EC cell lines treated with DMSO or three drugs as indicated. Representative data from three biological repeats was shown (mean ± SD). *P* values were calculated by unpaired two-sided Student’s *t*-test. Sulconazole, 50 μM; Menadione, 20 μM; GW8510, 15 μM. **e**, **g** Tumor growth in KYSE30 (**e**) and KYSE150 (**g**)-derived tumor xenograft mouse models treated with DMSO or three drugs as indicated. GW8510, 5 mg/kg; Menadione,10 mg/kg; Sulconazole, 10 mg/kg. **f**, **h** Growth curve of tumors as described in **e** and **g** (*n* = 5 independent animals). Data are presented as mean ± SD. *P* values were calculated by unpaired two-sided Student’s *t*-test.

To seek therapeutic strategies for EC patients, we selected proteins that were differentially expressed between tumor and non-tumor samples (BH adjusted *P* < 0.01, Wilcoxon signed-rank test, FC > 2 or < 0.5) for drug prediction. This led to the identification of 189 upregulated and 271 downregulated proteins in tumor samples (Supplementary Data 6a), which were used as the query signature and subsequently mapped to the Connectivity Map (CMAP)^48^ (Supplementary Figs. 6a, b). A high negative connectivity score indicated that the corresponding perturbagen could reverse the expression of the query signature. The top five candidate drugs with the highest negative connectivity scores from the mapped drugs are 1-(2,4-Dichlorobenzoyl)-1H-benzimidazole, HC toxin, chlorphenesin, cytochalasin B, and 2-Benzoylbenzene-1,4-diyl bis(4-bromo-3-nitrobenzoate) (Supplementary Fig. 6a, b and Supplementary Data 6b). We also searched therapeutic strategies more specific for patients with the S2 subtype, we selected proteins that were differentially expressed between both subtypes (BH adjusted *P* < 0.01, Wilcoxon signed-rank test, FC >1.5 or <0.67) and were significantly correlated with OS (Cox *P* < 0.05) for drug prediction. This led to the identification of 86 upregulated and 24 downregulated proteins in the S2 subtype (Supplementary Data 6c), which were used as the query signature and subsequently mapped to the Connectivity Map (CMAP) (Fig. 5a-b). We selected six candidate drugs with the highest negative connectivity scores from the mapped drugs for experimental verification in the current story (Fig. 5a and Supplementary Data 6d).

We first investigated the effects of the six drugs on six EC cell lines—KYSE30, KYSE150, KYSE450, TE1, TE3, and TE5—for cell viability. These six EC cell lines belong to the S2 subtype based on consensus clustering (Supplementary Fig. 7a). Meanwhile, immunoblotting analysis results revealed that ELOA and SCAF4 were highly expressed in these cell lines (Supplementary Fig. 7b). All six drugs could effectively inhibit the proliferation of all tested EC cell lines, with GW8510, Menadione, and Sulconazole being the most effective (Fig. 5c and Supplementary Data 6e). The effects of these three drugs on cell growth were further confirmed using a colony formation assay (Fig. 5d). Furthermore, to verify the effects of these drugs in mice, GW8510, menadione, or sulconazole were injected intraperitoneally into nude mice with xenograft tumors from KYSE30 and KYSE150 cells; tumor growth was markedly reduced (Fig. 5e–h).

**Fig. 6:**
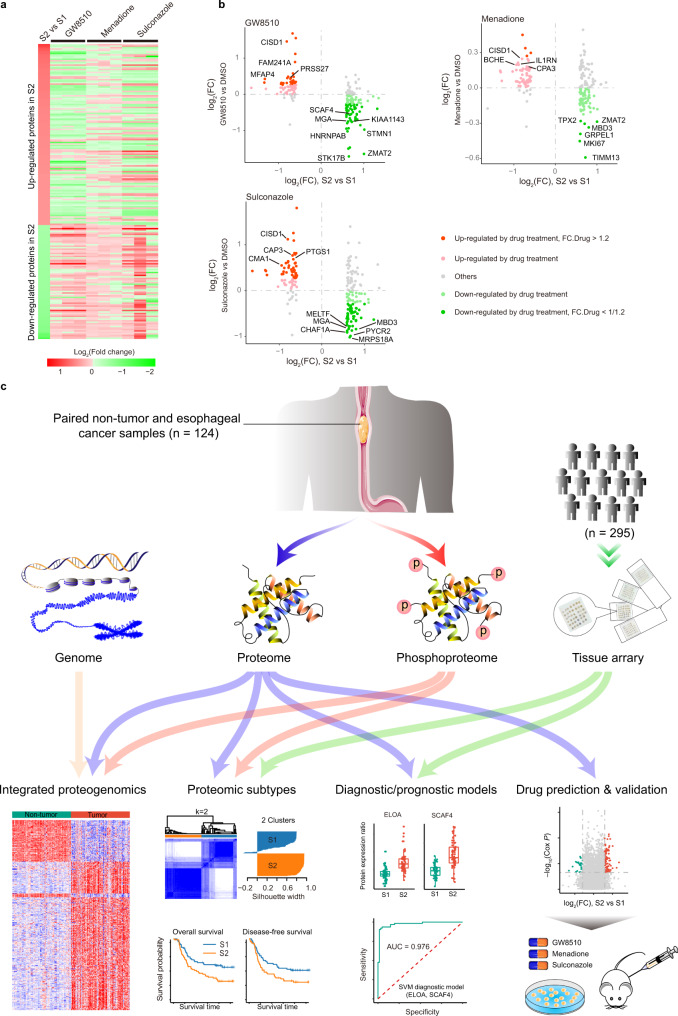
The effects of Sulconazole, Menadione and GW8510 on EC cell growth were linked to their regulation of differentially expressed proteins in the S1 and S2 subtypes. **a** The expression of proteins differentially expressed between S1 and S2 subtype in response to the three drugs as indicated in KYSE150 cells. Three biological repeats were performed. **b** Altered proteins in KYSE150 cells treated with three drugs. Light green indicates proteins that are up-regulated in S2 (*n* = 137) but downregulated by drug treatment. Light red indicates proteins that are downregulated in S2 (*n* = 93) but upregulated by drug treatment. Green and red indicate proteins that had fold change more than 1.2 in response to drug treatment. Other proteins are colored in gray. Representative subtype-risk proteins are marked. All *P* values were calculated by unpaired two-sided Student’s *t*-test. *P < 0.05, **P < 0.01, ***P < 0.001, ns, not significant. **c** Schematic of the proteomic analyses of esophageal cancer (EC). A large-scale, high-resolution mass spectrometry-based proteomic and phosphoproteomics profiling of paired non-tumor and esophageal cancer samples was reported. Genomic information and tissue arrays were integrated with the proteomic data for proteogenomic analysis, proteomic subtype definition, diagnostic/prognostic model construction, drug prediction, and validation. The analysis defined subtypes and subtype signature of EC, and provided molecular basis for finding potential treatments for EC.

To test if the effects of these three drugs on EC cell growth could be linked to their regulation of differentially expressed proteins in the S1 and S2 subtypes, proteomic analysis of KYSE150 cells following drug treatment was performed. Strikingly, all three drugs exhibited a remarkable impact on the vast majority of proteins that were differentially expressed in the S1 and S2 subtypes (Fig. 6a and Supplementary Data 6f–6i). Specifically, for the 124 upregulated proteins in S2, GW8510, Menadione, and Sulconazole treatment led to the downregulation of 95, 65, and 77 proteins, respectively. Meanwhile, for the 78 downregulated proteins in S2, 65, 69, and 67 proteins were activated in response to GW8510, Menadione, and Sulconazole treatment, respectively (Fig. 6b). The proteins altered by the three drugs contained a large number of subtype-risk proteins such as STK17B, ZMAT2, STMN1, TIMM13, MRPS18A, and CISD1, with 72, 52, and 59 proteins altered by GW8510, menadione, and sulconazole, respectively (Fig. 6b and Supplementary Data 6g–6i). These results suggested that GW8510, Menadione, and Sulconazole could serve as potential drugs for EC patients belonging to the S2 subtype by targeting dysregulated proteins in the S2 subtype.

## Discussion

Genomic analysis has broadened our understanding of the molecular events in EC^4–8,28^. Herein, global proteomic and phosphoproteomic analysis provided new insights into the biology of this malignancy. In addition to affirming some genomic alterations, the proteomic analysis has found alterations at the protein level that were inconsistent with the genomic analysis. Meanwhile, previously unknown alterations, such as increased protein expression and increased phosphorylation levels of numerous epigenetic regulators, were revealed, thus highlighting the importance of proteomic analysis to better understand EC.

Our quantitative proteomic analysis of paired tumor and non-tumor esophageal tissues revealed that the altered proteome in EC was highly similar to that in gastric cancer, including upregulated EMT, DNA replication, cell cycle, E2F targets, and G2/M checkpoint, and downregulated drug metabolism, fatty acid metabolism, and oxidative phosphorylation. This suggest that EC and gastric cancer pathogeneses may share common characteristics^15^, and analogous therapeutic approaches could therefore be considered. Moreover, phosphoproteomics revealed the hyperphosphorylation of many SR proteins in the spliceosome. Reversible phosphorylation regulates almost all functions of SR proteins, including interaction with other proteins, subnuclear localization, spliceosome assembly and disassembly, and nucleus-cytoplasm shuttling activity^26,27^. The hyperphosphorylation of SR proteins in tumor samples may lead to the misregulation of alternative splicing and eventually contribute to EC tumorigenesis. The mechanistic links between SR protein hyperphosphorylation and EC tumorigenesis warrant further investigation.

Unlike many other types of cancers, EC has no defined molecular subtypes to guide clinical treatment^3,6,21–23^. Here, we divided EC patients into two subtypes, a high-risk subtype S2 and a low-risk subtype S1, which could provide guidance for clinical treatment. Compared with the subtype S1, the subtype S2 exhibited expression profiling associated with stronger proliferation. The expression of proteins involved in E2F targets, G2/M checkpoint, and spliceosome gradually increased in the subtype S2 compared with the non-tumor, subtype S1 (Supplementary Fig. 4d), indicating that these proteins might account for the development of EC. Numerous proteins involved in ribosome and oxidative phosphorylation were specifically dysregulated in the subtype S2 (S2-specific proteins), indicating that these proteins might be related to the deterioration of EC. Although enhanced EMT has been often associated with tumor deterioration, EMT was enriched in the down-regulated proteins in the malignant S2 subtype, all of which were S2-specific. These proteins include TIMP3, ABI3BP, MYLK, and MYL9 (Supplementary Fig. 4d), whose downregulations have been reported to promote cell proliferation, invasion, and metastasis^40–43^. In contrast, most of these downregulated proteins were upregulated in the KYSE150 cells following GW8510, menadione, or sulconazole treatment (Supplementary Data 6g–i), suggesting that these S2-specific proteins may play an important roles in the development of EC. We also performed consensus clustering for the tumor samples based on proteins that are quantifiable with high confidence in at least half samples (Supplementary Fig. 2a, Prot 3, *n* = 9300). The top 25% of the most variable proteins (*n* = 2325) were selected for consensus clustering. Again, two major subtypes were identified that had maximal average silhouette width (0.69), which we named as S1-Prot3 and S2-Prot3 subtypes (Supplementary Fig. 8a–8c). It is worthy of noting that the average silhouette width based on Prot3 (0.69) was smaller than Prot5 (0.73). The S1-Prot3 and S2-Prot3 subtypes contained 35 and 89 tumor samples, respectively (Supplementary Fig. 8a). Strikingly, the tumor samples in S2 subtype we originally defined (*n* = 63) were all included in S2-Prot3 subtype (Supplementary Fig. 8d). 26 tumor samples in S1 subtype were now in S2-Prot3 subtype, but they clearly had lower consensus values (light blue) than other tumor samples in S2-Prot3 subtype (Supplementary Fig. 8a, d). Patients with the S2-Prot3 subtype had worse OS and disease-free survival (DFS) outcomes compared to those in the S1-Prot3 subtype (Supplementary Fig. 8e). Specifically, the OS median was 1338 and 920 days for S1-Prot3 and S2-Prot3 subtypes, respectively (log-rank *P* = 0.24), and the DFS median was 1215 and 655 days for S1-Prot3 and S2-Prot3 subtypes, respectively (log-rank *P* = 0.35) (Supplementary Fig. 8e). Altogether, the differentially expressed proteins and pathways in the two identified subtypes might help us to better understand the mechanisms underlying EC progression, and facilitate the identification of new therapeutic strategies.

Based on the differentially expressed proteins in tumor samples, we sought to identify potential drugs that may beneficial for EC patients. The top five candidate drugs with the highest negative connectivity scores from the mapped drugs are 1-(2,4-Dichlorobenzoyl)-1H-benzimidazole, HC toxin, chlorphenesin, cytochalasin B, and 2-Benzoylbenzene-1,4-diyl bis(4-bromo-3-nitrobenzoate) (Supplementary Fig. 6a, b and Supplementary Data 6b). 1-(2,4-dichlorobenzoyl)-1H-benzimidazole is a member of the class of benzamides obtained by the formal condensation of 1H-benzimidazole and 2,4-dichlorobenzoic acid. Benzimidazole derivatives have a variety of biological activities such as anticancer, antiviral, antibacterial, antifungal, antiparasitic, anti-inflammatory, proton pump inhibitors, anticoagulants, etc^49,50^. Both DNA and RNA viruses have been shown to activate the innate immunity in host cells through activating the cGAS/STING and RIG-I/MAVS signaling pathway, respectively, to induce the expression of type I interferons (IFNs) and type I interferon-stimulated genes (ISGs)^51–53^. A number of ISGs were found to be highly induced in tumor compared to non-tumor samples, such as MX1, IDO1, IFIT3, IFIT1, EPSTI1, GBP5, CAMP, GBP1, and OAS3 as shown in Fig. 2g and Supplementary Data 2a. Meanwhile, chronic fungal infection, *Fusobacterium nucleatum* (*F. nucleatum*) and *Porphyromonas gingivalis* infection, and activation of endogenous retroviruses have been linked to esophageal carcinogenesis and chemoresistance^54–56^. Therefore, whether the over-presentation of those ISGs in EC is linked to the above mentioned fungi, bacteria, and retroviruses, and the interferon signaling pathway could represent a targetable avenue for EC treatment remains as an interesting topic for future investigation. HC toxin is a potent, cell-permeable histone deacetylase (HDAC) inhibitor, which has certain inhibitory effects on breast cancer, colon cancer, and other tumors. In particular, HC toxin has a better anti-tumor effect in mature neuroblastoma than other HDAC inhibitors, which has been linked to cell cycle arrest, apoptosis induction and cell differentiation^57–59^. Chlorphenesin is an antigen-associated immunosuppressant that inhibits IgE-mediated histamine release. It is also used as an antimycotic agent^60,61^. Cytochalasin B is a cell-permeable mycotoxin binding to the barbed end of actin filaments, disrupting the formation of actin polymers^62,63^. 2-benzoylbenzene-1,4-diyl bis(4-bromo-3-nitrobenzoate) is a member of the class of benzophenones that is benzophenone substituted by (4-bromo-3-nitrobenzoyl)oxy groups at positions 1 and 4. It has been shown to have anticancer potential in breast and prostate cancer cells in vitro^64^. We also identified potential drugs that may specifically target patients belonging to the S2 subtype based on the differentially expressed proteins associated with subtype S2. Among them, menadione exhibited the most dramatic effects on cell proliferation. Menadione is a synthetic analog of vitamin K, which has been reported to exhibit anticancer activity against various types of cancers^65^. It can impair mitochondrial DNA replication and repair by inhibiting DNA polymerase gamma and induce reactive oxygen species-mediated apoptosis in human cancer cells^66^. Moreover, menadione has been shown to induce G2/M arrest in gastric cancer cells^67^ and G1 arrest in renal cell carcinoma^68^. This is consistent with the enrichment of DNA replication and repair and cell cycle-related pathways in proteins upregulated in EC. Importantly, menadione had minimal toxicity to human primary colon epithelial cells and non-tumorigenic HEK293 and HaCaT cells^69,70^. Another potential drug that may specifically target patients belonging to the S2 subtype was GW8510, which is a CDK2 (cyclin-dependent kinase 2) inhibitor. Drug repositioning found that it can be used to treat human colorectal cancer and lung squamous cell carcinoma by inhibiting RRM2^71,72^, an oncogenic protein in multiple cancer types^73–76^. We found that RRM2 was also upregulated in EC tumor samples. Meanwhile, GW8510 was not capable of inducing the apoptosis of normal human fibroblast^77^. Azole drugs, especially imidazole drugs, have shown antitumor efficacy in several cancer types, including lung cancer, prostate cancer, colon cancer, and breast cancer^78–82^. Sulconazole, a typical azole anti-fungal drug, is in the imidazole class and has been recently shown to inhibit cell proliferation, tumor growth, and cancer stem cell formation in human breast cancer stem cells^83^. However, the molecular mechanisms underlying the anticancer activity of azole drugs remain to be fully uncovered. Nevertheless, it has been reported to suppress invasion through the inhibition of MMP9^82^. Interestingly, MMP9 was upregulated in our EC tumor samples. The mechanism by which sulconazole inhibits the proliferation and migration of EC cells is also worthy of further study. The drugs we identified are potentially more specific to patients belonging to the subtype S2, and their clinical use might avoid the overtreatment of S1 patients. PTM-SEA analysis results revealed that the activity of several kinases including AMPKA2, PKCI, ST2, PDK1, DYRK2, CDC7, and DYRK1A was significantly upregulated in S2 subtype compared to S1 subtype. Testing the effects of inhibitors targeting these kinases on the growth of cells in S1 and S2 subtypes remains as an interesting topic for future investigation. Our study highlights the advantage of discovering drugs using the molecular subtypes defined by proteomic analysis.

In summary, our study revealed the dysregulated proteins and pathways in EC tumors, based on which two clinically relevant molecular subtypes, S1 and S2, were defined. Furthermore, diagnostic and prognostic subtype signatures composed of ELOA and SCAF4 were identified. We uncovered the differential proteome between the two subtypes and provided potential drugs, that can be utilized for the treatment of patients with the more aggressive S2 subtype.

## Methods

### Clinical sample acquisition

Paired tumor and adjacent non-tumor esophageal tissues from a cohort of 124 ESCC (esophageal squamous cell carcinoma) patients (Cohort 1, Supplementary Data 1a) were obtained from the Shantou Central Hospital. The adjacent non-tumor esophageal tissues were taken at least 5 cm away from the tumors. All the patients underwent curative resection from June 2011 to December 2013. The second independent cohort (Cohort 2) included 295 EC patients that underwent curative resection from November 2007 to January 2011 at the Shantou Central Hospital (Supplementary Data 5c). After resection, the tissue samples were snap-frozen in liquid nitrogen within 30 min and then stored in a −80 °C refrigerator before use. All the cases were classified according to the seventh edition of the American Joint Committee on Cancer (AJCC) pTNM system^84^. Clinical information on smoke, alcohol, and histopathological factors was obtained from the medical records. Overall survival (OS) was defined as the interval between surgery and death from tumors or between surgery and the last observation taken for surviving patients. Disease-free survival (DFS) was defined as the interval between surgery and diagnosis of relapse or death. Ethical approval was obtained from the ethical committee of the Central Hospital of Shantou City and the ethical committee of the Shantou University Medical College. Only resected samples from surgical patients with written informed consent were included.

### Cell lines and reagents

ESCC cell lines (KYSE30, KYSE150, and KYSE450) were established by Dr. Shimada Yutaka (Faculty of Medicine, Kyoto University, Kyoto, Japan)^85^. The TE cell lines (TE1, TE3, and TE5) were established by Dr. Nishihira (Institute of Development, Aging and Cancer, Tohoku University School of Medicine, Sendai, Japan)^86^. All cells were tested for mycoplasma contamination and cultured in RPMI-1640 medium supplemented with 10% FBS, penicillin (100 mg/mL) and streptomycin (100 mg/mL), respectively. All cells were maintained under the humidified 5% CO_2_ atmosphere at 37 °C. 8-Azaguanine (HY-B1468), Sulconazole nitrate (HY-B1460A), Menadione (HY-B0332), and Campathecin (HY-16560) were purchased from MedChem Express. GW8510 (sc-215122) was purchased from Santa Cruz Biotechnology. Thioguanine (S1774) was purchased from Selleck Chemicals.

### LC-MS/MS analysis

#### Protein extraction and digestion

The tissue and EC cell line samples were processed according to the Filter Aided Sample Preparation (FASP) method. The tryptic peptides were desalted by StageTips and lyophilized followed by labeling with TMT-11plex (Pierce) according to the manufacturer’s instructions. For the “internal reference” mixed sample used in TMT labeling, 60 pairs of tumor and adjacent non-tumor samples were randomly selected and mixed in equal protein amount. The peptides of mixed samples were divided into 35 µg per EP tube for each set of TMT labeling experiment as the internal reference. Two hundred microgram labeled peptides were off-line fractionated by bRP using a Waters XBridge BEH C18 5 μm 4.6 × 250 mm column (Waters) on an Ultimate 3000 high-pressure liquid chromatography (HPLC) system (Dionex) operating at 1 mL/min. Buffer A (5 mM ammonium formate) and buffer B (5 mM ammonium formate, 90% (v/v) ACN) were adjusted to pH 10 with ammonium hydroxide. Peptides were separated by a linear gradient from 5% B to 40% B in 90 min followed by a linear increase to 70% B in 6 min. A total of 96 fractions were collected. The 96 fractions were concatenated to 32 fractions, and all the peptide fractions were lyophilized.

#### TMT 11-plex labeling

The 124 paired tumor and non-tumor esophageal tissue samples were labeled in 25 groups of TMT 11-plex experiments for LC-MS/MS analysis. For each TMT 11-plex experiment, the mixed peptides were labeled with channel 131C as the internal reference, and five pairs of tumor and non-tumor esophageal tissue samples were labeled with the other ten channels (Tumor esophageal tissues labeled with 127N, 128N, 129N, 130N and 131N; non-tumor esophageal tissues labeled with 126, 127C, 128C, 129C, and 130C).

#### Phosphopeptide enrichment and fractionation

Three microgram protein of each selected samples were digested with Lys-C (1:100, w/w, Wako) for 6 h at 37 °C followed by trypsin (1:50, w/w, Promega) overnight at 37 °C. The resulting peptide mixture was acidified (pH 2–3) with formic acid, loaded onto Sep-Pak tC18 cartridges (Waters), desalted and eluted with 70% acetonitrile. The phosphopeptide enrichment was performed using High-Select Fe-NTA kit (Thermo Scientific, A32992) according to the manufacturer’s instructions. The eluates were collected for speed-vac and dried for fractionation. The Phospho-peptides were separated using high-pH reversed-phase chromatography (Hp-RP). In brief, the pipette tip was blocked using a layer of Empore 3M C8 disk and then filled with 5 mg of C18 reverse-phase medium (3 μm, Durashell, Agela Technologies). The tip was washed twice with 100 μL ACN and then with 100 μL 0.1% FA in water. The phosphopeptides were re-dissolved in 1% FA in water and loaded onto the tip and centrifuged at 1200 × *g* for 5 min to remove the liquid. Then, 100 μL water was loaded onto the tip and centrifuged at 1200 × *g* for 5 min to remove the liquid. The bound peptides were eluted with six gradients of elution buffer. The phosphopeptides were eluted with 100 μL of elution buffer 1 (15% ammonia) at 600 × *g* for 5 min, elution buffer 2 (15% ammonia, 2% ACN) at 900 × *g* for 5 min, elution buffer 3 (15% ammonia, 5% ACN) at 1100 × *g* for 5 min, elution buffer 4 (15% ammonia, 8% ACN) at 1400 × *g* for 5 min, elution buffer 5 (15% ammonia, 10% ACN) at 1400 × *g* for 5 min and elution buffer 6 (15% ammonia, 40% ACN) at 1400 × *g* for 5 min. The elutions were collected, and the fractions were combined into three fractions as follows: fraction 1 with 6, fraction 2 with 4 and fraction 3 with 5. Thus, three final fractions of eluted phosphopeptides were obtained and instantaneously dried in a SpeedVac concentrator at 45 °C, and stored at −80 °C before use^16^.

#### Proteomic LC-MS/MS analysis

TMT MS experiments were performed on a nanoscale EASY-nLC 1200UHPLC system or nanoU3000UHPLC system (Thermo Fisher Scientific) connected to an Orbitrap Fusion Lumos equipped with a nanoelectrospray source (Thermo Fisher Scientific). Mobile phase A contained 0.1% formic acid (v/v) in water; mobile phase B contained 0.1% formic acid in 80% acetonitrile (ACN). The peptides were dissolved in 0.1% formic acid (FA) with 2% ACN and separated on a RP-HPLC analytical column (75 μm × 25 cm) packed with 2 μm C18 beads (Thermo Fisher Scientific) using a linear gradient ranging from 9 to 32% ACN in 100 min and followed by a linear increase to 50% B in 20 min at a flow rate of 300 nL/min. The Orbitrap Fusion Lumos acquired data in a data-dependent manner alternating between full-scan MS and MS2 scans. The spray voltage was set at 2.2 kV and the temperature of ion transfer capillary was 300 °C. The MS spectra (350−1500 *m*/*z*) were collected with 60,000 resolution, AGC of 4 × 10^5^ and 50 ms maximal injection time. Selected ions were sequentially fragmented in a 3 s cycle by HCD with 38% normalized collision energy, specified isolated windows 0.7 *m*/*z*, 50,000 resolution. AGC of 1 × 10^5^ and 105 ms maximal injection time were used. Dynamic exclusion was set to 30 s. Unassigned ions or those with a charge of 1+ and >7+ were rejected for MS/MS.

#### Phosphoproteomic LC-MS/MS analysis

For phosphoproteomic analysis, The MS system was the same as above. The spray voltage was set at 2.2 kV and the temperature of ion transfer capillary was 300 °C. The MS spectra (350−1500 *m*/*z*) were collected with 120,000 resolution, AGC of 4 × 10^5^ and 50 ms maximal injection time. Selected ions were sequentially fragmented in a 3 s cycle by HCD with 30% normalized collision energy, specified isolated windows 1.6 *m*/*z*, 30,000 resolution. AGC of 5 × 10^4^ and 80 ms maximal injection time were used. Dynamic exclusion was set to 30 s. Unassigned ions or those with a charge of 1+ and >7+ were rejected for MS/MS.

#### MS data analysis

The data were collected using Xcalibur software (Thermo Fisher Scientific, version 3.0). Raw data were processed using Proteome Discoverer (PD, version 2.2), and MS/MS spectra were searched against the reviewed SwissProt human proteome database. All searches were carried out with precursor mass tolerance of 20 ppm, fragment mass tolerance of 0.02 Da, oxidation (Met) (+15.9949 Da), TMT6plex (Lys) (229.163 Da) and acetylation (protein N-terminus) (+42.0106 Da) as variable modifications, carbamidomethylation (Cys) (+57.0215 Da), TMT6plex (N-terminal) (229.163 Da) as fixed modification and three trypsin missed cleavages allowed. In EC phosphorylation data analysis, phospho (STY) was chosen as a variable modification. Only peptides with at least six amino acids in length were considered. The peptide and protein identifications were filtered by PD to control the false discovery rate (FDR) <1%. At least one unique peptide was required for protein identification.

### Global proteomic data analysis

#### Data normalization

The protein expression ratio was calculated as the ratio of the sample abundance to the abundance of the “internal reference” mixed sample. To mitigate systematic, sample-specific bias in the quantification of protein levels^12^, the protein expression ratios were log_2_-transformed and normalized using the mean centering method across all proteins. In the normalized samples, the proteins should have a log_2_-transformed expression ratio centered at zero.

#### Data filtering

The proteomic data was filtered to five datasets at different levels according to the following criteria (Supplementary Fig. 2a). (1) Dataset 1 (Prot1) included all proteins that quantified in at least one of the 25 TMT groups. (2) For dataset 2 (Prot2), proteins were required to be quantified in high confidence in at least one of the 25 TMT groups. (3) Dataset 3 (Prot3) included proteins that quantified with high confidence in at least half samples. (4) Dataset 4 (Prot4) included proteins quantified in all 124 paired samples. (5) Dataset 5 (Prot5) included proteins quantified with high confidence in all 124 paired samples.

#### Batch effect analysis

The unsupervised principle component analysis (PCA) and hierarchical clustering were performed on protein expression ratios of common proteins in Prot5 to assess the batch effect due to TMT multiplexes in R v.3.6.2. The assessment is mainly based on two variables: sample class (Tumor and non-tumor) and group (Batch 1–25). For PCA, the leading PCs of the protein expression data well separated the tumor from non-tumor samples, and the samples in the same group did not clustered together, indicating that there is no obvious batch effect. The R package ggbiplot v.0.55 was used to show the confidence intervals. For hierarchical clustering, we used the complete linkage algorithm with Euclidean distance as the distance measure. Samples with high similarity tend to cluster together. If the samples are clustered together predominantly depending on sample classes rather than sample groups, the batch effect is negligible relative to biological differences. The hierarchical clustering was performed using the R package pheatmap v.1.0.12.

#### Tumor versus non-tumor differential proteomic analysis

Tumor versus non-tumor differential proteomic analysis was performed on 9300 proteins that are quantified in at least half samples with high confidence (Prot3). The Wilcoxon signed-rank test was performed on overlapping samples to assess the statistical significance *P* value of protein expression difference between paired tumor and non-tumor samples. Proteins with BH adjusted *P* value < 0.01 and fold change (FC.Prot, expressed as ratio of average protein expression ratio between tumor and non-tumor samples) >1.5 or <0.67 were considered to be significantly upregulated or downregulated proteins in tumor samples (Supplementary Data 2a).

#### Correlation between protein expression ratios and clinical outcome

We used two methods to evaluate the association between protein expression ratios and patient risk. (1) Cox PH model. A univariate Cox PH model was used to estimate the hazard ratio (HR), confidence interval, and Cox *P* value of each protein. HR >1 means that the expression of the protein is positively correlated with patient risk, while HR <1 means a negative correlation. The correlation is considered significant if Cox *P* value < 0.05. Correlation with OS and DFS are estimated separately. (2) The X-tile method^87^. The EC patients were divided into two groups according to the expression values of the protein by the X-tile method. A log-rank *P* value is calculated by a log-rank test on survival difference of the two groups. For each protein, the protein expression is considered to be positively correlated with patient risk, if the average survival time of the group with high protein expression is shorter than that of the group with low protein expression, and vice versa. The correlation is considered significant if log-rank *P* value < 0.05. The X-tile method was used to estimate the correlation between protein expression and OS in this study.

#### EC-associated risk proteins

A protein is defined as an EC-associated risk protein if it meets the following two criteria: (1) The protein is significantly upregulated or downregulated in tumor samples compared to non-tumor samples (FC.Prot >1.5 or <0.67, and BH adjusted *P* value < 0.01); (2) The protein expression is significantly correlated with OS according to the X-tile method (log-rank *P* value < 0.05).

### Phosphoproteomic data analysis

#### Quantification and normalization of phosphosites

The expression abundance of each phosphopeptide was determined by the sum of the three final fractions of eluted phosphopeptides, and the phosphosite abundance was determined by the median abundance for all phosphopeptides matching that site. The expression abundance of the phosphosites was subjected to quantile normalization^88^ implemented in the R package limma v.3.42.2. Missing values were imputed with the minimum value across the phosphoproteomic data.

#### Phosphoproteomic data filtering

The phosphoproteomic data was filtered to three datasets at different levels according to the following criteria (Supplementary Fig. 2g). (1) Dataset 1 (Phos1) included 73,651 highly reliable phosphosites (FDR < 1%). (2) Dataset 2 (Phos2) included 67,393 phosphosites with quantified values. (3) Dataset 3 (Phos3) included 61,471 phosphosites that quantified in at least half samples.

#### Tumor vs. non-tumor differential phosphoproteomic analysis

Tumor vs. non-tumor differential phosphoproteomic analysis was performed on 61,471 phosphosites that quantified in at least half samples (Phos3). The Wilcoxon signed-rank test was performed on overlapping samples to assess the statistical significance *P* value of phosphosite abundance difference between paired tumor and non-tumor samples. Phosphosites with BH adjusted *P* value < 0.01 and fold change (FC.Phos, expressed as ratio of average phosphosite abundance between tumor and non-tumor samples) >2 or <0.5 were considered to be significantly upregulated or downregulated phosphosites in tumor samples (Supplementary Data 2c). The proteins with significantly upregulated or downregulated phosphosites in tumor samples are considered as differential phosphoproteins in tumor vs. non-tumor samples.

### Proteomic subtyping analysis

#### Consensus clustering for proteomic data

The normalized protein expression ratios of 124 tumor samples in Prot5 were used to identify molecular subtypes in EC using the consensus clustering method^89^ implemented in the R package ConsensusClusterPlus v.1.50.0. The proteins were sorted according to their coefficients of variation, and the top 25% of the most variant proteins (1617) were selected for consensus clustering. The consensus clustering was implemented using the following detailed settings: maximum cluster number (maxK) = 6, number of repeats (reps) = 1000, proportion of items to sample (pItem) = 0.8, proportion of features to sample (pFeature) = 0.8, cluster algorithm (clusterAlg) = “hc” (hierarchical clustering), and distance=“spearman”. The optimal number of clustering was determined by the average silhouette width, which was calculated using the R package cluster v.2.1.0. The average silhouette width for *k* = 2 was larger than *k* = 3, 4, 5, and 6 (Fig. 3b). Thus, the EC patients were finally clustered into two molecular subtypes S1 and S2.

#### Correlation between molecular subtype and clinical outcome

Kaplan–Meier curves and log-rank tests were used to evaluate the OS and DFS difference between two molecular subtypes S1 and S2. A univariant Cox PH model was used to evaluate the prognostic power of molecular subtype (*S*1 = 1/*S*2 = 2) on OS and DFS, respectively (Supplementary Data 4b). The independence between molecular subtypes and clinicopathological factors (Age, Gender (Female = 0/Male = 1), Smoke (No = 0/Yes = 1), Alcohol (No = 0/Yes = 1), and pTNM stage (I = 1/II = 2/III = 3)) was estimated by a multivariate Cox PH model. A significance level of 0.05 was used.

#### Correlations between molecular subtype and clinicopathologic factors

The correlation between molecular subtypes and clinicopathologic factors was examined by *χ*^2^-test or Fisher’s exact test for categorical variables, and Wilcoxon rank-sum test for continuous variables (Supplementary Data 4c).

#### S2 vs. S1 differential proteomic analysis

Proteomic subtype S2 vs. S1 differential proteomic analysis was performed on 6468 common proteins that are quantified with high confidence in all 124 paired patients (Prot5). The Wilcoxon rank-sum test was performed to assess the statistical significance *P* value of protein expression difference between S2 and S1. Proteins with BH adjusted *P* value < 0.01 and fold change (FC.Prot, expressed as ratio of average protein expression ratio between S2 and S1) >1.5 or <0.67 were considered to be significantly upregulated or downregulated proteins in samples belonging to S2 (Supplementary Data 4d).

#### S2 vs. S1 differential phosphoproteomic analysis

Subtype S2 vs. S1 differential phosphoproteomic analysis was performed on 61,471 phosphosites that quantified in at least half samples (Phos3). The Wilcoxon rank-sum test was performed to assess the statistical significance *P* value of phosphosite abundance difference between S2 and S1. Phosphosites with *P* value < 0.01 and fold change (FC.Phos, expressed as ratio of average phosphosite abundance between S2 and S1) >2 or <0.5 were considered to be significantly upregulated or downregulated phosphosites in samples belonging to S2 (Supplementary Data 4e). The proteins with significantly upregulated or downregulated phosphosites in samples belonging to S2 are considered as differential phosphoproteins in S2 vs. S1 subtypes.

#### Subtype-risk proteins

A protein is defined as a subtype-risk protein if it meets the following two criteria: (1) The protein is significantly upregulated or downregulated in S2 subtype compared to S1 (FC.Prot >1.5 or <0.67, and BH adjusted *P* value < 0.01); (2) The protein expression is significantly correlated with OS (Cox *P* value < 0.05).

### Functional enrichment analysis

Functional enrichment analysis was performed using Metascape^90^ to infer dysregulated KEGG pathways, Hallmark gene sets, and GO biological functions that enriched on differential protein sets, including (1) upregulated or downregulated proteins in tumor vs. non-tumor samples (Supplementary Data 2d); (2) upregulated or downregulated phosphoproteins in tumor vs. non-tumor samples (Supplementary Data 2e); (3) upregulated or downregulated proteins in S2 vs. S1 subtype (Supplementary Data 4f); and (4) upregulated or downregulated phosphoproteins in S2 vs. S1 subtype (Supplementary Data 4g). The statistical significance *P* value was evaluated by hypergeometric test, and adjusted by BH correction. Significant calls were obtained on the basis of a BH adjusted *P* value (*q* value) cut-off of 0.05.

### PTM-SEA analysis

PTM signatures database (PTMsigDB, v1.9.0) was first downloaded from http://prot-shiny-vm.broadinstitute.org:3838/ptmsigdb-app/. The implementation of PTM-SEA on GitHub was used (https://github.com/broadinstitute/ssGSEA2.0). The following parameters were used to run PTM-SEA. sample.norm.type: rank; weight: 0.75; statistic: area.under.RES; output.score.type: NES; nperm: 1000; min.overlap: 5; correl.type: z.score.

### Tissue microarrays (TMAs) and immunohistochemistry (IHC)

TMAs construction and IHC staining were based on standard techniques as previously described^91^. TMAs were constructed using 295 tumor esophageal tissues from Cohort 2. The clinical information of the 295 EC patients was provided in Supplementary Data 5c. Markers that were used in this study included ELOA, SCAF4, MX1, OAS3, and IFIT1. Rabbit anti-ELOA polyclonal antibody (NBP1-87040, Novus), anti-SCAF4 polyclonal antibody (PA5-36611, Thermo fisher), and anti-MX1 polyclonal antibody (13750-1-AP, Proteintech) were diluted at 1:200. Rabbit anti-OAS3 polyclonal antibody (21915-1-AP, Proteintech) and mouse anti-IFIT1 monoclonal antibody (TA500948S, Origene) were diluted at 1:50. Immunostaining was performed by an automated quantitative pathology imaging system (Perkin Elmer, Waltham, MA, USA)^92^. Firstly, we used the Vectra 2.0.8 for automated image acquisition, and obtained the color images. Secondly, the spectral libraries were built by the Nuance 3.0 software. The color images were then evaluated by inform 1.2 software following three steps: (1) Segmented tumor region from the tissue compartments; (2) Segmented cells from the tumor region; and (3) Calculated *H*-score based on the optical density. *H*-score (= (% at 0) * 0 + (% at 1+) * 1 + (% at 2+) * 2 + (% at 3+) * 3) produces a continuous protein expression value in the range of 0–300.

### Western blotting

Laemmli sample buffer (Bio-Rad, Hercules, CA, USA) was used to lyse cells and extract total protein. The total protein was resolved by sodium dodecyl sulfate polyacrylamide gel electrophoresis (SDS-PAGE) and then transferred onto polyvinylidene fluoride membrane. The membrane was blocked with 5% skimmed milk powder diluted with Tris-buffered saline Tween-20. Rabbit anti-ELOA polyclonal antibody (NBP1-87040, NOVUS) and anti-SCAF4 polyclonal antibody (PA5-36611, Thermo Fisher) were diluted at 1:1000. β-Actin monoclonal antibody (66009-1-Ig, Proteintech) was diluted at 1:5000. Immunoblotting was imaged by Chemodoc MP (Bio-Rad, Hercules, CA, USA) system.

### Subtype diagnostic model

#### Signature identification

To identify signature proteins that classify molecular subtypes, the differential proteins in S2 vs. S1 subtype were used as the initial feature set for signature identification. Feature selection was implemented on the initial feature set using the R package mlr v.2.17.0. In order to facilitate clinical utility, we limit the maximum number of features to no more than 4. Support vector machine (SVM) was used as the classifier because it is characterized by good generalization ability. Other parameters were set as follows: feature selection method = “sfs” (sequential forward search), resampling algorithm = “Subsample”, number of resampling = 50, and performance measures = “auc”. We set the maximum number of features (“max.features”) to 1, 2, 3, or 4, and repeated the feature selection process 100 times. Feature combinations that are frequently identified during feature selection are considered as robust signatures (Supplementary Data 5a).

#### Signature evaluation

Five-fold cross-validation was used to evaluate the classification performance of each signature. The 124 EC samples in Cohort 1 were randomly split into five subsets with equal size. Four subsets were used as the training set to train the SVM model for each signature, and the remaining one subset was used as the test set. The performance of the signature was measured by evaluating its AUC and accuracy on the test set. Each subset was used in turn as the test set. For an unbiased evaluation, we repeated the above process 100 times. The average AUC and accuracy of the resulting 500 AUCs and accuracies were reported to evaluate the overall predictive performance of the signature (Supplementary Data 5b).

#### Construction of subtype diagnostic model

The signature 4 (ELOA and SCAF4; Supplementary Data 5b) was selected as the subtype diagnostic signature. We performed *z*-score transformation for the protein expression of each protein so that the mean value of the protein expression on all 124 tumor samples is 0, and the variance is 1. Using the SVM model implemented in the R package mlr v.2.17.0, the subtype diagnostic model was constructed based on the normalized protein expression of ELOA and SCAF4 and the known molecular subtypes of the 124 EC patients in Cohort 1.

#### Subtype prediction

For 295 EC patients in Cohort 2, the *H*-scores of ELOA and SCAF4 were log_2_-transformed and further normalized by *z*-score transformation. For each patient, the normalized *H*-scores of ELOA and SCAF4 were subjected to the subtype diagnosis model, and the probability that the patient belongs to subtype S1 or S2 was predicted. For EC cell lines, the protein expression ratios of ELOA and SCAF4 quantified by TMT 11-plex were normalized by *z*-score transformation and then subjected to the subtype diagnosis model for subtype prediction. The patient or EC cell line was classified into the subtype with greater probability.

### Subtype prognostic model

#### “pTNM+Subtype” model

As molecular subtype and pTNM stage are independent prognostic factors (Supplementary Data 4b), we combined the two factors to improve the prognostic power of the pTNM staging system. For OS, a Cox PH model was constructed using the molecular subtype and pTNM stage as independent variables and the OS information as dependent variable (referred to as “pTNM+Subtype” model) based on the EC patients in Cohort 1. The risk score for OS of a new patient can be predicted based on the molecular subtype and pTNM stage of the patient by the “pTNM+Subtype” model. For DFS, the “pTNM+Subtype” model was constructed in the same way except using the DFS information as dependent variable.

#### “pTNM+Subtype 3c” model

The “pTNM+Subtype 3c” model was an EC staging system based on the “pTNM+Subtype” model. The EC patients in Cohort 1 were clustered into three groups using k-means clustering (*k* = 3) on the risk scores predicted by the “pTNM+Subtype” model. The three groups of EC patients were classified as low-risk, medium-risk, and high-risk according to their average risk scores from low to high. A new patient will be predicted to be low-risk, medium-risk, or high-risk based on its risk score using the nearest neighbor method.

#### Evaluation of subtype prognostic model

As clinical outcomes are time dependent, we used time-dependent ROC curve (TDROC) for censored data and AUC^93^ implemented in the R package survcomp v.1.36.1 to evaluate the predictive performance of prognostic models. Larger AUC at time *t* indicates better predictability of time to event (patient risk) at time *t*. We plotted AUCs ranging from 1 to 7 years to compare the overall predictive performance of prognostic models at any time *t* ranging from 1 to 7 years.

### CMAP-based drug prediction

We first constructed query signatures and then mapped the query signatures to CMAP^48^. CMAP is a resource that uses transcriptional expression data from cultured human cells treated with perturbagens to probe relationships between diseases, cell physiology, and therapeutics. Each reference gene-expression profile in CMAP is represented as a rank-ordered gene list. The query signature is compared to each rank-ordered list to determine whether upregulated proteins tend to appear near the top of the list and downregulated proteins near the bottom (“positive connectivity”) or vice versa (“negative connectivity”), yielding a “connectivity score” ranging from +1 to −1. To predict candidate drugs for EC patients, the proteins that were differentially expressed between tumor and non-tumor samples (BH adjusted *P* < 0.01, Wilcoxon signed-rank test, FC >2 or <0.5) were selected as the query signature. To predict candidate drugs for patients with the S2 subtype, the proteins that meet the following two conditions were selected as the query signature: (1) The protein expression is upregulated (FC.Prot > 1.5) or downregulated (FC.Prot < 0.67) in subtype S2; and (2) The protein expression is significantly correlated with OS (Cox *P* value < 0.05). The connectivity score of each perturbagen was calculated using the query signature. We sorted perturbagens according to their connectivity scores in increasing order. As a high negative connectivity score indicates that the corresponding perturbagen reversed the expression of the query signature, the top drugs with the highest negative connectivity scores were predicted as potential drugs.

### Cell viability assay (MTS assay)

The cells were digested and cultured in a medium containing 10% serum to form a single cell suspension. The cells were counted and inoculated into 96-well plates with 10,000 cells per well. The volume of the medium per well was 100 μL. The cells were cultured for 12 h to adhere to the cell wall, and then the culture medium with different drug concentration was changed. After 24 h of continuous culture, 20 μL MTS (G3581, Promega) was added into each well, and then incubation was continued for 2 h. The absorbance value of each pore was measured at the wavelength of 492 nm selected by MK3 enzyme scale of Thermo Scientific company. The cell viability at different drug concentrations was calculated and then mapped with GraphPad prism 7 software.

### Colony formation assay

In the 12-well plates, 500 cells per well were inoculated. After the cells adhered to the wall, the corresponding concentration of drug was added to the cell culture medium for 24 h. The culture medium was replaced with the normal one for 2 weeks. After washing with 4 °C precooled PBS, the culture was fixed with 4 °C precooled methanol and glacial acetic acid 3:1 for 20 min and stained with crystal violet for 15 min. The colony was photographed with the ChemiDoc Touch (Bio-Rad) and the colony numbers was calculated with Image J software (US National Institutes of Health, Bethesda, MD, USA). Each experiment was made in triplicate.

### Xenograft assay in nude mice

Animal experiments were carried out according to the program approved by the Medical Animal Care and Welfare Committee of Shantou University Medical College. Nude mice (Vital River Laboratories Animal Technology, Beijing, China) aged 3–5 weeks were randomly divided into four groups. KYSE30 (2 × 10^6^) and KYSE150 (1 × 10^6^) cells were injected into the armpit of mice, respectively. Drugs injection began when the average volume of tumor reached 50mm^3^. GW8510 (5 mg/kg), menadione (10 mg/kg) and sulconazole (10 mg/kg) were injected intraperitoneally every 3 days. The tumor volume was measured every 3 days and calculated according to the following formula (width^2^ × length)/2. The tumor was resected and weighed after the mice were euthanized with excessive CO_2_ 30 days after inoculation. The feeding conditions were specific pathogen free animal laboratory with 28 °C and 50% humidity, providing sufficient water and diet.

### Reporting summary

Further information on research design is available in the Nature Research Reporting Summary linked to this article.

## Supplementary information

Supplementary Information

Description of Additional Supplementary Files

Supplementary Data 1

Supplementary Data 2

Supplementary Data 3

Supplementary Data 4

Supplementary Data 5

Supplementary Data 6

Reporting Summary

## Data Availability

The data that support the findings of this study—including clinical information, and proteome and phosphoproteome data—are available within the paper and its Supplementary Information. The raw files of proteome and phosphoproteome datasets can be obtained from PRIDE database (accession number PXD021701)^94^ or iProX database (accession number IPX0002501000)^95^. PTM signatures database (PTMsigDB, v1.9.0) was downloaded from http://prot-shiny-vm.broadinstitute.org:3838/ptmsigdb-app/. Source data are provided with this paper.

## Source data

Source Data

## References

[1] Bray F (2018). Global cancer statistics 2018: GLOBOCAN estimates of incidence and mortality worldwide for 36 cancers in 185 countries. Cancer J. Clin..

[2] Chen W (2016). Cancer statistics in China, 2015. Cancer J. Clin..

[3] Pennathur A, Gibson MK, Jobe BA, Luketich JD (2013). Oesophageal carcinoma. Lancet.

[4] Song Y (2014). Identification of genomic alterations in oesophageal squamous cell cancer. Nature.

[5] Lin DC (2014). Genomic and molecular characterization of esophageal squamous cell carcinoma. Nat. Genet..

[6] Cancer Genome Atlas Research Network. (2017). Integrated genomic characterization of oesophageal carcinoma. Nature.

[7] Zhang L (2015). Genomic analyses reveal mutational signatures and frequently altered genes in esophageal squamous cell carcinoma. Am. J. Hum. Genet..

[8] Cui Y (2020). Whole-genome sequencing of 508 patients identifies key molecular features associated with poor prognosis in esophageal squamous cell carcinoma. Cell Res..

[9] Zhang H (2016). Integrated proteogenomic characterization of human high-grade serous ovarian cancer. Cell.

[10] Zhang B (2014). Proteogenomic characterization of human colon and rectal cancer. Nature.

[11] Clark DJ (2019). Integrated proteogenomic characterization of clear cell renal cell carcinoma. Cell.

[12] Vasaikar S (2019). Proteogenomic analysis of human colon cancer reveals new therapeutic opportunities. Cell.

[13] Mun DG (2019). Proteogenomic characterization of human early-onset gastric cancer. Cancer Cell.

[14] Mertins P (2016). Proteogenomics connects somatic mutations to signalling in breast cancer. Nature.

[15] Ge S (2018). A proteomic landscape of diffuse-type gastric cancer. Nat. Commun..

[16] Jiang Y (2019). Proteomics identifies new therapeutic targets of early-stage hepatocellular carcinoma. Nature.

[17] Gao Q (2019). Integrated proteogenomic characterization of HBV-related hepatocellular carcinoma. Cell.

[18] Xu JY (2020). Integrative proteomic characterization of human lung adenocarcinoma. Cell.

[19] Chen YJ (2020). Proteogenomics of non-smoking lung cancer in East Asia delineates molecular signatures of pathogenesis and progression. Cell.

[20] Gillette MA (2020). Proteogenomic characterization reveals therapeutic vulnerabilities in lung adenocarcinoma. Cell.

[21] Cancer Genome Atlas Research Network. (2014). Comprehensive molecular characterization of gastric adenocarcinoma. Nature.

[22] Curtis C (2012). The genomic and transcriptomic architecture of 2000 breast tumours reveals novel subgroups. Nature.

[23] Cancer Genome Atlas Research Network. (2012). Comprehensive molecular portraits of human breast tumours. Nature.

[24] Yang H, Sukocheva O, Hussey D, Watson D (2012). Estrogen, male dominance and esophageal adenocarcinoma: Is there a link?. World J. Gastroenterol..

[25] Sukocheva, O., Wee, C., Ansar, A., Hussey, D. & Watson, D. Effect of estrogen on growth and apoptosis in esophageal adenocarcinoma cells. *Dis. esophagus* **26**, 628–635 (2012). 10.1111/dote.12000 23163347

[26] Ma X, He F (2003). Advances in the study of SR protein family. Genomics Proteom. Bioinform..

[27] Stamm S (2008). Regulation of alternative splicing by reversible protein phosphorylation. J. Biol. Chem..

[28] Sawada G (2016). Genomic Landscape of esophageal squamous cell carcinoma in a Japanese population. Gastroenterology.

[29] Hao J-J (2016). Spatial intratumoral heterogeneity and temporal clonal evolution in esophageal squamous cell carcinoma. Nat. Genet..

[30] Gao Y-B. et al. Genetic landscape of esophageal squamous cell carcinoma. *Nat. Genet.* **46**, 1097–1102 (2014). 10.1038/ng.3076 25151357

[31] Cheng C (2016). Whole-genome sequencing reveals diverse models of structural variations in esophageal squamous cell carcinoma. Am. J. Hum. Genet..

[32] Lin DC, Wang MR, Koeffler HP (2018). Genomic and epigenomic aberrations in esophageal squamous cell carcinoma and implications for patients. Gastroenterology.

[33] Fogal V, Hsieh J-K, Royer C, Zhong S, Lu X (2005). Cell cycle-dependent nuclear retention of p53 by E2F1 requires phosphorylation of p53 at Ser315. EMBO J..

[34] Katayama H (2004). Sen SPhosphorylation by aurora kinase A induces Mdm2-mediated destabilization and inhibition of p53. Nat. Genet..

[35] Rubin S (2013). Deciphering the retinoblastoma protein phosphorylation code. Trends Biochem. Sci..

[36] Loeser H (2017). Somatic BRCA1-associated protein 1 (BAP1) loss is an early and rare event in esophageal adenocarcinoma. Mol. Clin. Oncol..

[37] Mori T (2015). Somatic alteration and depleted nuclear expression of BAP1 in human esophageal squamous cell carcinoma. Cancer Sci..

[38] Guan Y (2020). Downregulating integrin subunit alpha 7 (ITGA7) promotes proliferation, invasion, and migration of papillary thyroid carcinoma cells through regulating epithelial-to-mesenchymal transition. Acta Biochim. Biophys. Sin..

[39] Bhandari A (2018). ITGA7 functions as a tumor suppressor and regulates migration and invasion in breast cancer. Cancer Manag. Res..

[40] Su C-W, Lin C-W, Yang W-E, Yang S-F (2019). TIMP-3 as a therapeutic target for cancer. Ther. Adv. Med. Oncol..

[41] Anania MC (2011). TIMP3 regulates migration, invasion and in vivo tumorigenicity of thyroid tumor cells. Oncogene.

[42] Latini FRM, Hemerly JP, Oler G, Riggins GJ, Cerutti JM (2008). Re-expression of ABI3-binding protein suppresses thyroid tumor growth by promoting senescence and inhibiting invasion. Endocr. Relat. Cancer.

[43] Tan X, Chen M (2014). MYLK and MYL9 expression in non-small cell lung cancer identified by bioinformatics analysis of public expression data. Tumour Biol..

[44] Aso T, Lane WS, Conaway JW, Conaway RC (1995). Elongin (SIII): a multisubunit regulator of elongation by RNA polymerase II. Science.

[45] Gregersen LH (2019). SCAF4 and SCAF8, mRNA anti-terminator proteins. Cell.

[46] Fliedner A (2020). Variants in SCAF4 cause a neurodevelopmental disorder and are associated with impaired mRNA processing. Am. J. Hum. Genet..

[47] Heagerty PJ, Lumley T, Pepe MS (2000). Time-dependent ROC curves for censored survival data and a diagnostic marker. Biometrics.

[48] Subramanian A (2017). A next generation connectivity map: L1000 platform and the first 1,000,000 profiles. Cell.

[49] Vasava MS (2020). Benzimidazole: a milestone in the field of medicinal chemistry. Mini Rev. Med. Chem..

[50] Ganie AM, Dar AM, Khan FA, Dar BA (2019). Benzimidazole derivatives as potential antimicrobial and antiulcer agents: a mini review. Mini Rev. Med. Chem..

[51] Barrat FJ, Crow MK, Ivashkiv LB (2019). Interferon target-gene expression and epigenomic signatures in health and disease. Nat. Immunol..

[52] Michalska A, Blaszczyk K, Wesoly J, Bluyssen HAR (2018). A positive feedback amplifier circuit that regulates interferon (IFN)-stimulated gene expression and controls type I and type II IFN responses. Front. Immunol..

[53] Schoggins JW, Rice CM (2011). Interferon-stimulated genes and their antiviral effector functions. Curr. Opin. Virol..

[54] Zhu F (2017). Autoreactive T cells and chronic fungal infection drive esophageal carcinogenesis. Cell Host Microbe.

[55] Liu Y (2021). Fusobacterium nucleatum confers chemoresistance modulating autophagy oesophageal squamous cell carcinoma. Br. J. Cancer.

[56] Gao, S. et al. Porphyromonas gingivalis infection exacerbates oesophageal cancer and promotes resistance to neoadjuvant chemotherapy. *Br. J. Cancer* **12**, 1–12 (2021). 10.1038/s41416-021-01419-5 PMC8329259 33981017

[57] Joung KE, Kim DK, Sheen YY (2004). Antiproliferative effect of trichostatin A and HC-toxin in T47D human breast cancer cells. Arch. Pharm. Res..

[58] Kamitani H (2001). Expression of 15-lipoxygenase-1 is regulated by histone acetylation in human colorectal carcinoma. Carcinogenesis.

[59] Deubzer HE (2008). Anti-neuroblastoma activity of Helminthosporium carbonum (HC)-toxin is superior to that of other differentiating compounds in vitro. Cancer Lett..

[60] Lichtenstein LM, Adkinson NF (1969). Chlorphenesin: a new inhibitor IgE-mediated histamine release. J. Immunol..

[61] Thoma K, Kübler N, Reimann E (1997). The photostability of antimycotics. 3. Photostability of locally acting antimycotics. Die Pharmazie.

[62] Chang HT (2016). Mechanisms underlying effect of the mycotoxin cytochalasin B on induction of cytotoxicity, modulation of cell cycle, Ca(2+) homeostasis and ROS production in human breast cells. Toxicology.

[63] Hwang J (2013). Cytochalasin B induces apoptosis through the mitochondrial apoptotic pathway in HeLa human cervical carcinoma cells. Oncol. Rep..

[64] Saidi L (2019). Synthesis of benzophenones and in vitro evaluation of their anticancer potential in breast and prostate. Cancer Cells.

[65] Chlebowski RT, Dietrich M, Akman S, Block JB (1985). Vitamin K3 inhibition of malignant murine cell growth and human tumor colony formation. Cancer Treat. Rep..

[66] Sasaki R (2008). DNA polymerase gamma inhibition by vitamin K3 induces mitochondria-mediated cytotoxicity in human cancer cells. Cancer Sci..

[67] Lee MH (2016). Menadione induces G2/M arrest in gastric cancer cells by down-regulation of CDC25C and proteasome mediated degradation of CDK1 and cyclin B1. Am. J. Transl. Res..

[68] Degen M, Alexander B, Choudhury M, Eshghi M, Konno S (2013). Alternative therapeutic approach to renal-cell carcinoma: induction of apoptosis with combination of vitamin K3 and D-fraction. J. Endourol..

[69] Suresh S, Raghu D, Karunagaran D (2013). Menadione (Vitamin K3) induces apoptosis of human oral cancer cells and reduces their metastatic potential by modulating the expression of epithelial to mesenchymal transition markers and inhibiting migration. Asian Pac. J. Cancer Prev..

[70] Kishore C, Sundaram S, Karunagaran D (2019). Vitamin K3 (menadione) suppresses epithelial-mesenchymal-transition and Wnt signaling pathway in human colorectal cancer cells. Chemico-Biol. Interact..

[71] Hsieh YY, Chou CJ, Lo HL, Yang PM (2016). Repositioning of a cyclin-dependent kinase inhibitor GW8510 as a ribonucleotide reductase M2 inhibitor to treat human colorectal cancer. Cell Death Discov..

[72] Chen P (2018). Gemcitabine resistance mediated by ribonucleotide reductase M2 in lung squamous cell carcinoma is reversed by GW8510 through autophagy induction. Clin. Sci..

[73] Li C (2018). RRM2 promotes the progression of human glioblastoma. J. Cell. Physiol..

[74] Liu X, Peng J, Zhou Y, Xie B, Wang J (2019). Silencing RRM2 inhibits multiple myeloma by targeting the Wnt/β‑catenin signaling pathway. Mol. Med. Rep..

[75] Sun H (2019). RRM2 is a potential prognostic biomarker with functional significance in glioma. Int. J. Biol. Sci..

[76] Mazzu YZ (2019). A novel mechanism driving poor-prognosis prostate cancer: overexpression of the DNA repair gene, ribonucleotide reductase small subunit M2 (RRM2). Clin. Cancer Res..

[77] Dong F (2006). Downregulation of XIAP and induction of apoptosis by the synthetic cyclin-dependent kinase inhibitor GW8510 in non-small cell lung cancer cells. Cancer Biol. Ther..

[78] Taplin ME (2009). Phase II study of androgen synthesis inhibition with ketoconazole, hydrocortisone, and dutasteride in asymptomatic castration-resistant prostate cancer. Clin. Cancer Res..

[79] Aftab BT, Dobromilskaya I, Liu JO, Rudin CM (2011). Itraconazole inhibits angiogenesis and tumor growth in non-small cell lung cancer. Cancer Res..

[80] Antonarakis ES (2013). Repurposing itraconazole as a treatment for advanced prostate cancer: a noncomparative randomized phase II trial in men with metastatic castration-resistant prostate cancer. Oncologist.

[81] Kadavakollu S, Stailey C, Kunapareddy CS, White S (2014). Clotrimazole as a cancer drug: a short review. Med. Chem..

[82] Bae SH, Park JH, Choi HG, Kim H, Kim SH (2018). Imidazole antifungal drugs inhibit the cell proliferation and Invasion of human breast cancer cells. Biomol. Ther..

[83] Choi, H. S., Kim, J. H., Kim, S. L. & Lee, D. S. Disruption of the NF-κB/IL-8 signaling axis by sulconazole inhibits human breast cancer stem cell formation. *Cells* **8**, 1007 (2019). 10.3390/cells8091007 PMC6770215 31480284

[84] Rice T, Blackstone E, Rusch V (2010). 7th Edition of the AJCC cancer staging manual: esophagus and esophagogastric junction. Ann. Surg. Oncol..

[85] Shimada Y, Imamura M, Wagata T, Yamaguchi N, Tobe T (1992). Characterization of 21 newly established esophageal cancer cell lines. Cancer.

[86] Nishihira T, Hashimoto Y, Katayama M, Mori S, Kuroki T (1993). Molecular and cellular features of esophageal cancer cells. J. Cancer Res. Clin. Oncol..

[87] Camp RL (2004). X-Tile: a new bio-informatics tool for biomarker assessment and outcome-based cut-point optimization. Clin. Cancer Res..

[88] Bolstad BM, Irizarry R, Astrand M, Speed TP (2003). A comparison of normalization methods for high density oligonucleotide array data based on bias and variance. Bioinformatics.

[89] Hayes DN (2010). ConsensusClusterPlus: a class discovery tool with confidence assessments and item tracking. Bioinformatics.

[90] Yingyao, Z. et al. Metascape provides a biologist-oriented resource for the analysis of systems-level datasets. *Nat. commun.* **10**, 1–10 (2019). 10.1038/s41467-019-09234-6 PMC6447622 30944313

[91] Xie J-J (2011). Prognostic implication of ezrin expression in esophageal squamous cell carcinoma. J. Surg. Oncol..

[92] Liu W (2018). MASAN: a novel staging system for prognosis of patients with oesophageal squamous cell carcinoma. Br. J. Cancer.

[93] Heagerty P, Lumley T, Pepe M (2000). Time-dependent ROC curves for censored survival data and a diagnostic marker. Biometrics.

[94] Perez-Riverol Y (2019). The PRIDE database and related tools and resources in 2019: improving support for quantification data. Nucleic Acids Res..

[95] Ma J (2019). iProX: an integrated proteome resource. Nucleic Acids Res..

