# Supplementary material for: Large-scale and high-resolution mass spectrometry-based proteomics profiling defines molecular subtypes of esophageal cancer for therapeutic targeting
Source: Nat Commun. 2021 Aug 16;12:4961. doi: 10.1038/s41467-021-25202-5 (PMC8368010; doi:10.1038/s41467-021-25202-5)
Supplement: Supplementary file 1 — Supplementary Information [file 41467_2021_25202_MOESM1_ESM.pdf]

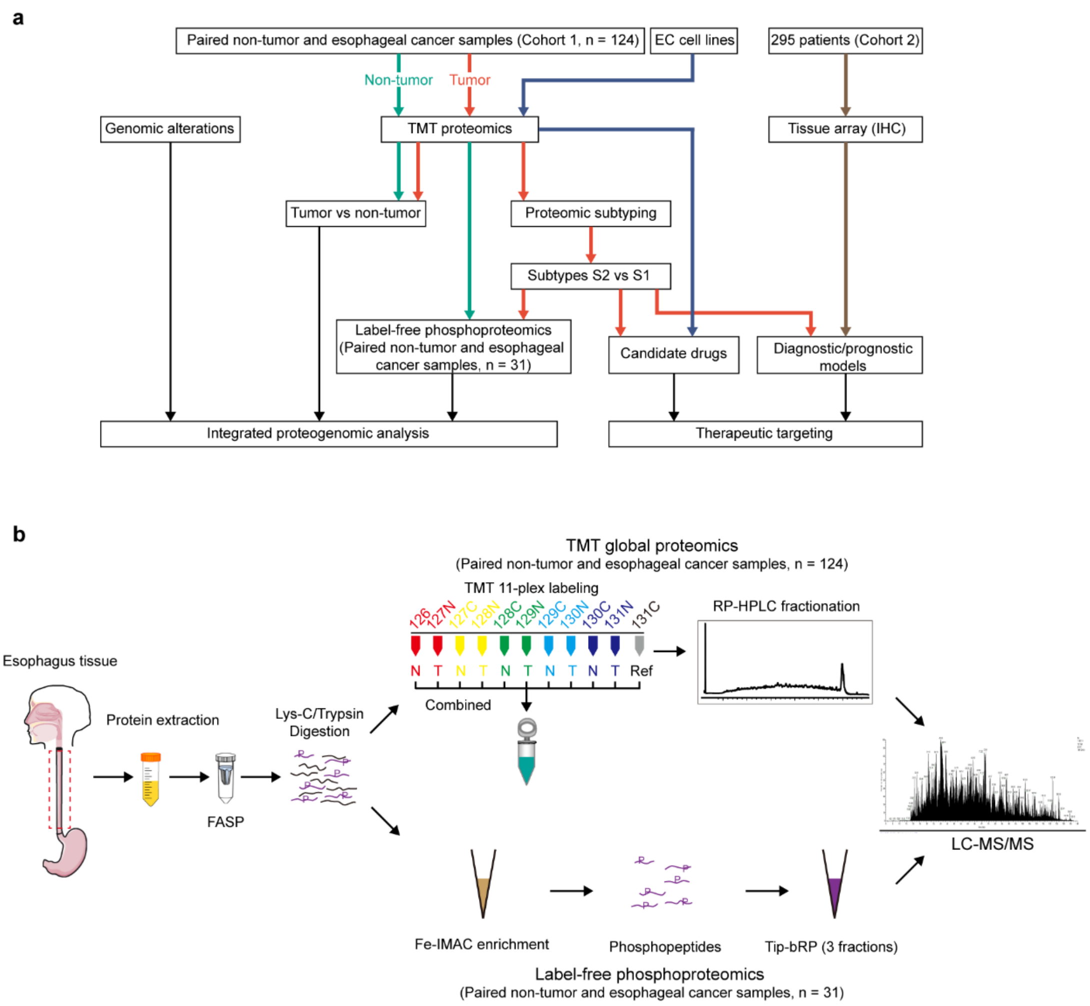

Supplementary Figure 1. An overview of TMT proteomics and label-free phosphoproteomics. (a) Workflow of the EC proteomic study. 124 paired tumor and non-tumor esophageal tissues (Cohort 1) were subjected to TMT proteomic analysis, of which 31 paired tissues were selected for label-free phosphoproteomic analysis. EC genomic alterations from the literature were collected for integrated proteogenomic analysis. Molecular subtype, S1 and S2, and subtype diagnosis model was defined based on proteomic analysis. Furthermore, molecular subtype was combined with immunohistochemistry of 295 patients (Cohort 2) to validate subtype diagnostic model. Potential drugs for treating patients in subtype S2 were predicted based on differentially expressed proteins between subtype S1 and S2. EC cell lines were used to validate the therapeutic effects of candidate drugs. (b) Workflow of TMT 11-plex proteomic and label-free phosphoproteomic analysis. EC tumor and non-tumor tissues were subjected to dry pulverization, protein extraction and trypsin digestion. For TMT 11-plex quantification, a total of 248 tumor and non-tumor esophagus tissues from 124 patients were analyzed in 25 TMT 11-

plex experiments with 5 paired tumor and adjacent non-tumor tissues and the internal reference sample. The reference sample contained 60 pairs of tumor and adjacent non-tumor tissues mixed in equal protein amount. The labeled peptides were combined for RP-HPLC fractionation. For label-free phosphoproteome quantification, 62 tumor and non-tumor esophagus tissues from 31 patients were analyzed.

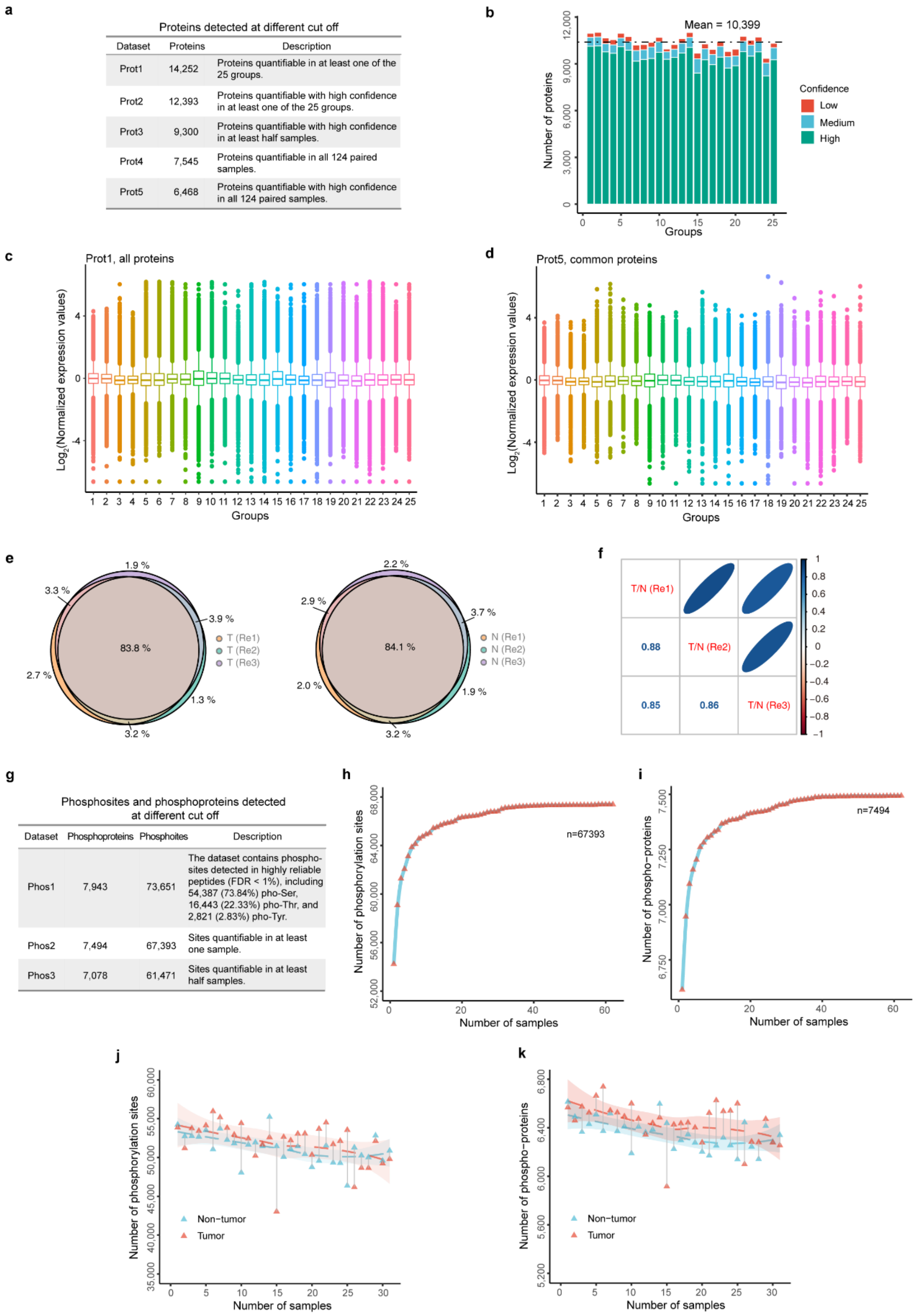

Supplementary Figure 2. Quality assessment for TMT proteomic and label-free phosphoproteomic data.

(a) Proteins detected by proteomic analysis at different levels as indicated. (b) The distribution of the number of proteins with different confidence levels identified in each group of samples.

12,393 (86.96%) proteins were quantified with high confidence. (c) Distribution of protein expression ratios (tumor vs non-tumor) of 14,252 proteins in the Prot1 dataset (n = 10125, 10704, 11026, 11170, 11384, 11503, 11560, 11606, 11704, 11788, 11856, 11902, 11969, 12113, 12140, 12169, 12188, 12231, 12243, 12256, 12298, 12338, 12372, 12378, 12393 for the groups 1-25, respectively). (d) Distribution of protein expression ratios (tumor vs non-tumor) of 6,468 proteins in the Prot5 dataset (n = 6,468 for each group). In the box plots (c and d), the middle bar represents the median, and the box represents the interquartile range; bars extend to 1.5× the interquartile range. (e) Phosphorylation sites detected by label-free phosphorproteomics experiment in triplicates for one pair of EC tumor and the adjacent non-tumor tissues are shown by venn diagram. (f) Pearson correlation coefficiency of the intensity of phosphopeptides identified in biological replicates of the pair of EC tumor and the adjacent non-tumor tissues as shown in (e). (g) Phosphorylation sites and phosphoproteins detected by phosphoproteomic analysis at different levels as indicated. (h) Cumulative number of phosphosites quantified as a function of the number of samples. (i) Cumulative number of phosphoproteins quantified as a function of the number of samples. (j) Number of phosphosites quantified in tumor (red) and non-tumor (blue) samples. The paired samples are annotated by grey lines. The dashed curves with 95% confidence intervals were fitted by local polynomial regression. The number of phosphosites quantified in tumor samples are larger than those in non-tumor samples (Mean: 51942 versus 51203; $P = 0.047$, one-sided Wilcox signed rank test). (k) Number of phosphoproteins quantified in tumor (red) and non-tumor (blue) samples. The paired samples are annotated by grey lines. The dashed curves with 95% confidence intervals were fitted by local polynomial regression. The number of phosphoproteins quantified in tumor samples are larger than those in non-tumor samples (Mean: 6,447 versus 6,349; $P = 0.001$, one-sided Wilcox signed rank test).

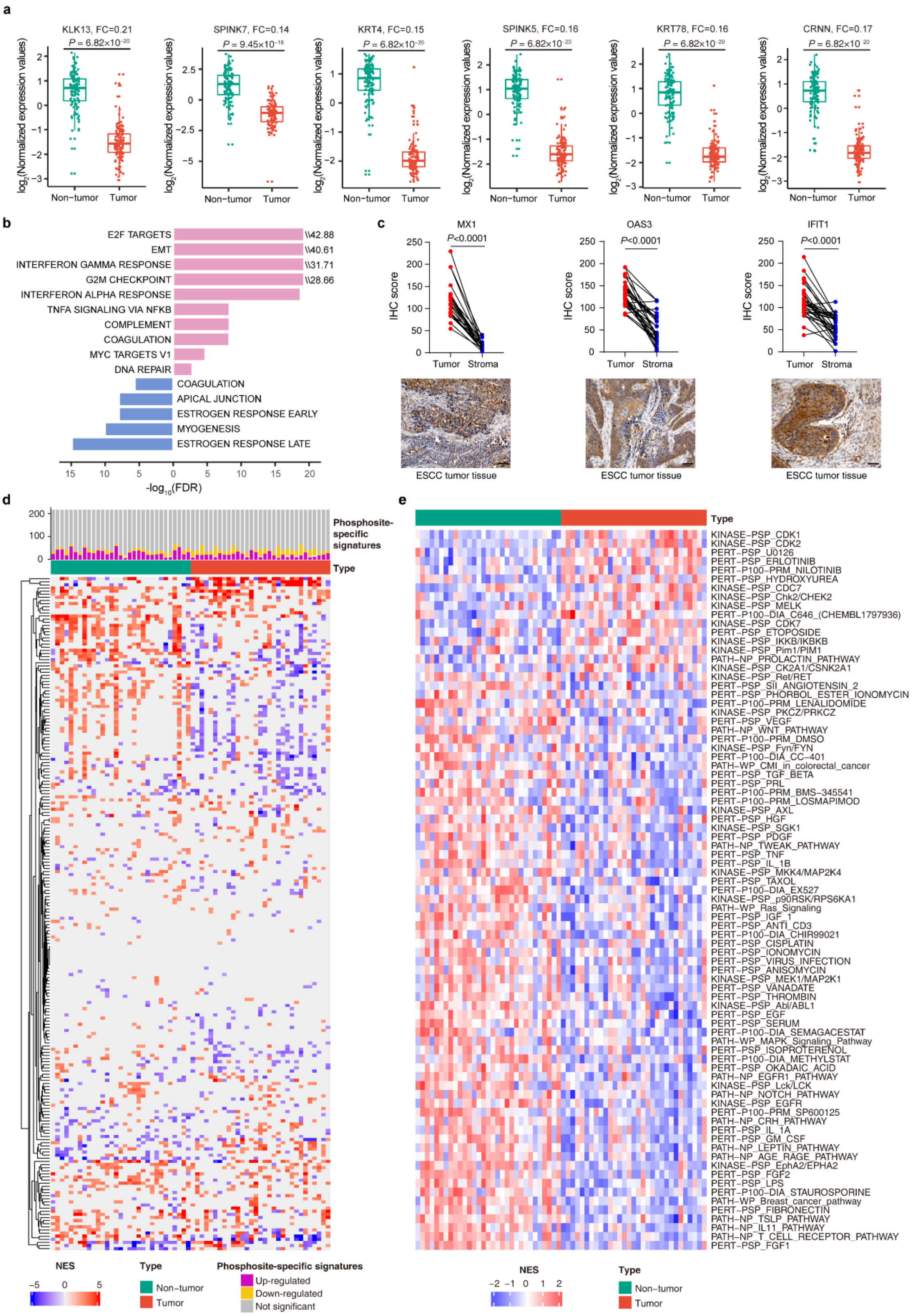
a
KLK13, FC=0.21
P = 6.82×10^-20
SPINK7, FC=0.14
P = 9.45×10^-16
KRT4, FC=0.15
P = 6.82×10^-20
SPINK5, FC=0.16
P = 6.82×10^-20
KRT78, FC=0.16
P = 6.82×10^-20
CRNN, FC=0.17
P = 6.82×10^-20
log2(Normalized expression values)
Non−tumor
Tumor
b
E2F TARGETS
EMT
INTERFERON GAMMA RESPONSE
G2M CHECKPOINT
INTERFERON ALPHA RESPONSE
TNFA SIGNALING VIA NFKB
COMPLEMENT
COAGULATION
MYC TARGETS V1
DNA REPAIR
42.88
40.61
31.71
28.66
COAGULATION
APICAL JUNCTION
ESTROGEN RESPONSE EARLY
MYOGENESIS
ESTROGEN RESPONSE LATE
-log10(FDR)
c
MX1
OAS3
IFIT1
P<0.0001
IHC score
Tumor
Stroma
ESCC tumor tissue
d
Phosphosite-specific signatures
Type
NES
Non−tumor
Tumor
Phosphosite-specific signatures
Up-regulated
Down-regulated
Not significant
e
Type
KINASE−PSP_CDK1
KINASE−PSP_CDK2
PERT−PSP_U0126
PERT−PSP_ERLOTINIB
PERT−P100−PRM_NILOTINIB
PERT−PSP_HYDROXYUREA
KINASE−PSP_CDC7
KINASE−PSP_Chk2/CHEK2
KINASE−PSP_MELK
PERT−P100−DIA_C646_(CHEMBL1797936)
KINASE−PSP_CDK7
PERT−PSP_ETOPOSIDE
KINASE−PSP_IKKB/IKBKB
KINASE−PSP_Pim1/PIM1
PATH−NP_PROLACTIN_PATHWAY
KINASE−PSP_CK2A1/CSNK2A1
KINASE−PSP_Ret/RET
PERT−PSP_SII_ANGIOTENSIN_2
PERT−PSP_PHORBOL_ESTER_IONOMYCIN
PERT−P100−PRM_LENALIDOMIDE
KINASE−PSP_PKCZ/PRKCZ
PERT−PSP_VEGF
PATH−NP_WNT_PATHWAY
PERT−P100−PRM_DMSO
KINASE−PSP_Fyn/FYN
PERT−P100−DIA_CC−401
PATH−WP_CML_in_colorectal_cancer
PERT−PSP_TGF_BETA
PERT−PSP_PRL
PERT−P100−PRM_BMS−345541
PERT−P100−PRM_LOSMAPIMOD
KINASE−PSP_AXL
PERT−PSP_HGF
KINASE−PSP_SGK1
PERT−PSP_PDGF
PATH−NP_TWEAK_PATHWAY
PERT−PSP_TNF
PERT−PSP_IL_1B
KINASE−PSP_MKK4/MAP2K4
PERT−PSP_TAXOL
PERT−P100−DIA_EX527
KINASE−PSP_p90RSK/RPS6KA1
PATH−WP_Ras_Signaling
PERT−PSP_IGF_1
PERT−PSP_ANTI_CD3
PERT−P100−DIA_CHIR99021
PERT−PSP_CISPLATIN
PERT−PSP_IONOMYCIN
PERT−PSP_VIRUS_INFECTION
PERT−PSP_ANISOMYCIN
KINASE−PSP_MEK1/MAP2K1
PERT−PSP_VANADATE
PERT−PSP_THROMBIN
KINASE−PSP_Abl/ABL1
PERT−PSP_EGF
PERT−PSP_SERUM
PERT−P100−DIA_SEMAGACESTAT
PATH−WP_MAPK_Signaling_Pathway
PERT−PSP_ISOPROTERENOL
PERT−P100−DIA_METHYLSTAT
PERT−PSP_OKADAIC_ACID
PATH−NP_EGFR1_PATHWAY
KINASE−PSP_Lck/LCK
PATH−NP_NOTCH_PATHWAY
KINASE−PSP_EGFR
PERT−P100−PRM_SP600125
PATH−NP_CRH_PATHWAY
PERT−PSP_IL_1A
PERT−PSP_GM_CSF
PATH−NP_LEPTIN_PATHWAY
PATH−NP_AGE_RAGE_PATHWAY
KINASE−PSP_EphA2/EPHA2
PERT−PSP_FGF2
PERT−PSP_LPS
PERT−P100−DIA_STAUROSPORINE
PATH−WP_Breast_cancer_pathway
PERT−PSP_FIBRONECTIN
PATH−NP_TSLP_PATHWAY
PATH−NP_IL11_PATHWAY
PATH−NP_T_CELL_RECEPTOR_PATHWAY
PERT−PSP_FGF1
NES
Type
Non−tumor
Tumor

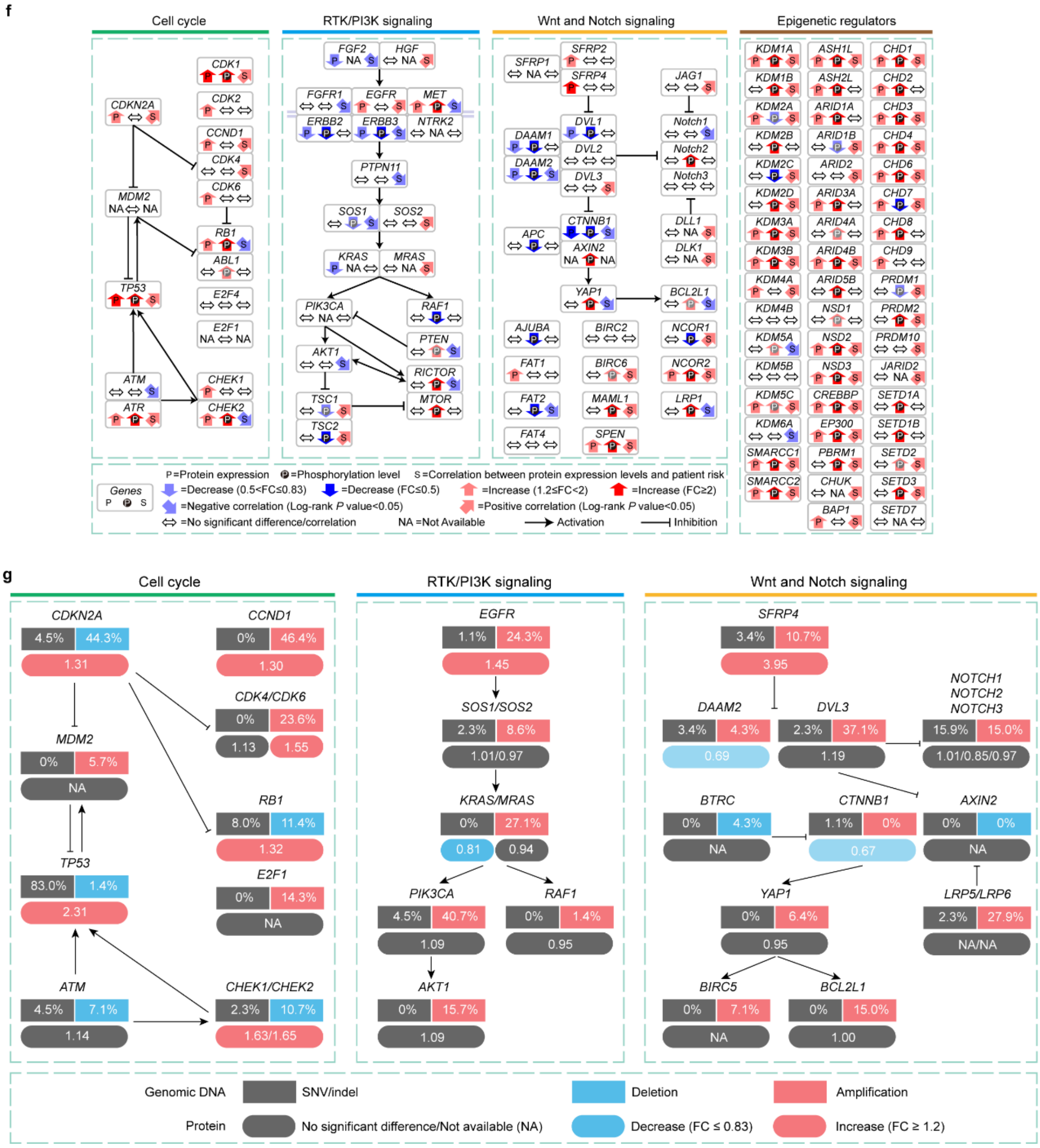
f
Cell cycle
RTK/PI3K signaling
Wnt and Notch signaling
Epigenetic regulators
CDK1
CDK2
CDKN2A
CCND1
CDK4
CDK6
MDM2
RB1
ABL1
TP53
E2F4
E2F1
ATM
ATR
CHEK1
CHEK2
FGF2
HGF
FGFR1
EGFR
MET
ERBB2
ERBB3
NTRK2
PTPN11
SOS1
SOS2
KRAS
MRAS
PIK3CA
RAF1
PTEN
AKT1
RICTOR
TSC1
TSC2
MTOR
SFRP1
SFRP2
SFRP4
JAG1
DVL1
DVL2
DVL3
DAAM1
DAAM2
Notch1
Notch2
Notch3
APC
CTNNB1
AXIN2
DLL1
DLK1
YAP1
BCL2L1
AJUBA
BIRC2
NCOR1
FAT1
BIRC6
NCOR2
FAT2
MAML1
LRP1
FAT4
SPEN
KDM1A
KDM1B
KDM2A
KDM2B
KDM2C
KDM2D
KDM3A
KDM3B
KDM4A
KDM4B
KDM5A
KDM5B
KDM5C
KDM6A
SMARCC1
SMARCC2
ASH1L
ASH2L
ARID1A
ARID1B
ARID2
ARID3A
ARID4A
ARID4B
ARID5B
NSD1
NSD2
NSD3
CREBBP
EP300
PBRM1
CHUK
BAP1
CHD1
CHD2
CHD3
CHD4
CHD6
CHD7
CHD8
CHD9
PRDM1
PRDM2
PRDM10
JARID2
SETD1A
SETD1B
SETD2
SETD3
SETD7
Genes
P = Protein expression
P = Phosphorylation level
S = Correlation between protein expression levels and patient risk
= Decrease (0.5<FC≤0.83)
= Decrease (FC≤0.5)
= Increase (1.2≤FC<2)
= Increase (FC≥2)
= Negative correlation (Log-rank P value<0.05)
= Positive correlation (Log-rank P value<0.05)
⇔ = No significant difference/correlation
NA = Not Available
Activation
Inhibition
g
Cell cycle
RTK/PI3K signaling
Wnt and Notch signaling
CDKN2A
4.5% 44.3%
1.31
CCND1
0% 46.4%
1.30
CDK4/CDK6
0% 23.6%
1.13 1.55
MDM2
0% 5.7%
NA
RB1
8.0% 11.4%
1.32
TP53
83.0% 1.4%
2.31
E2F1
0% 14.3%
NA
ATM
4.5% 7.1%
1.14
CHEK1/CHEK2
2.3% 10.7%
1.63/1.65
EGFR
1.1% 24.3%
1.45
SOS1/SOS2
2.3% 8.6%
1.01/0.97
KRAS/MRAS
0% 27.1%
0.81 0.94
PIK3CA
4.5% 40.7%
1.09
RAF1
0% 1.4%
0.95
AKT1
0% 15.7%
1.09
SFRP4
3.4% 10.7%
3.95
DAAM2
3.4% 4.3%
0.69
DVL3
2.3% 37.1%
1.19
NOTCH1
NOTCH2
NOTCH3
15.9% 15.0%
1.01/0.85/0.97
BTRC
0% 4.3%
NA
CTNNB1
1.1% 0%
0.67
AXIN2
0% 0%
NA
YAP1
0% 6.4%
0.95
LRP5/LRP6
2.3% 27.9%
NA/NA
BIRC5
0% 7.1%
NA
BCL2L1
0% 15.0%
1.00
Genomic DNA
SNV/indel
Deletion
Amplification
Protein
No significant difference/Not available (NA)
Decrease (FC ≤ 0.83)
Increase (FC ≥ 1.2)

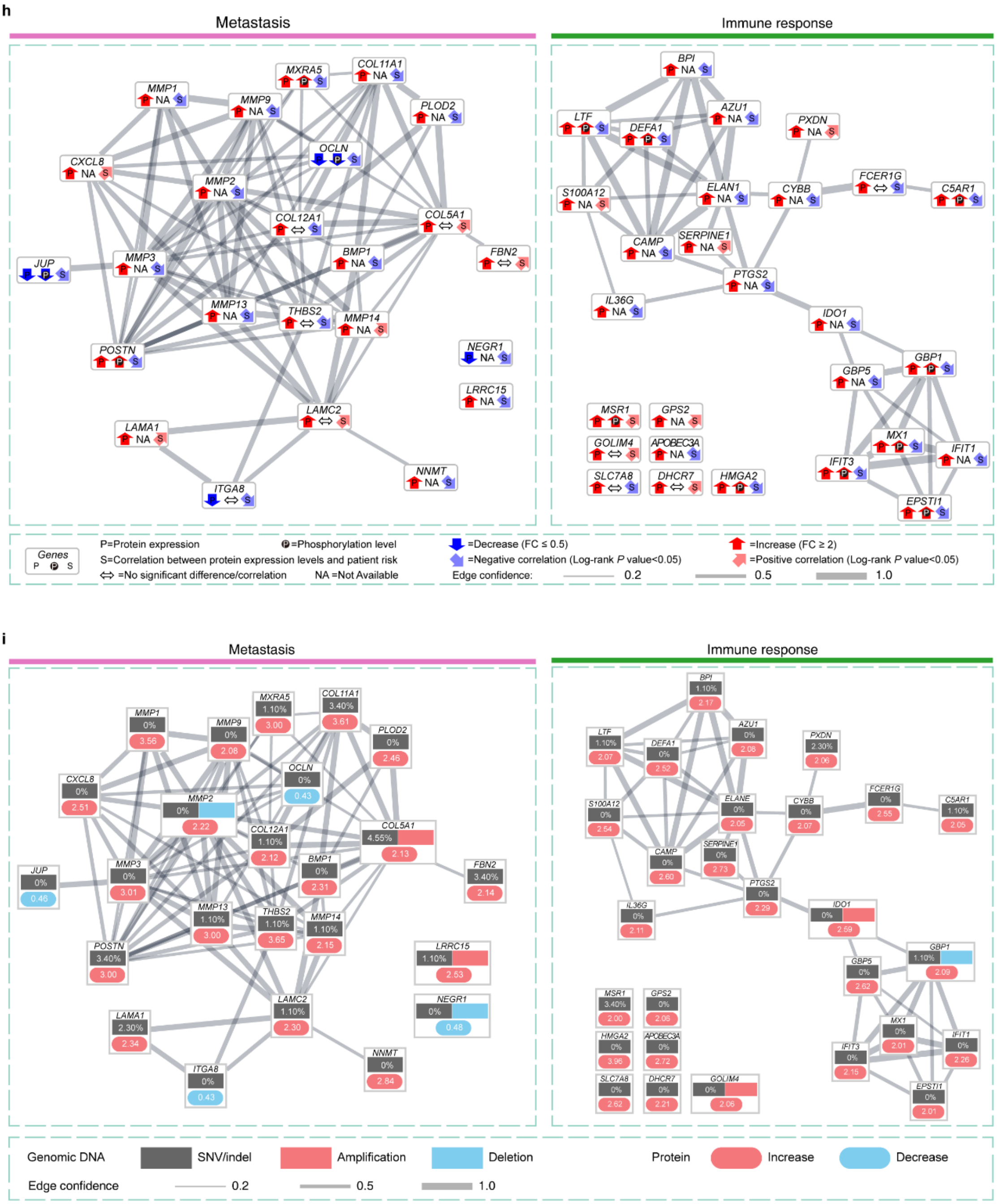

Supplementary Figure 3. Dysregulated proteins, hallmark gene sets and pathways in EC detected by proteomic analysis.

(a) The expression of esophageal-specific proteins as indicated is shown by box plots (n = 124). *P* values were calculated by two-sided Wilcoxon signed-rank test. The middle bar represents the median, and the box represents the interquartile range; bars extend to 1.5× the interquartile range. (b) Hallmark get sets enriched for differentially expressed proteins as shown in Fig. 2a. Pink and blue bars indicate pathways enriched by up- and down-regulated proteins, respectively. (c) Immunohistochemistry (IHC) score for three interferon-stimulated genes (ISGs)

in tumor and corresponding stroma cells is shown (MX1, n = 22; OAS3, n = 31; IFIT1, n=32). Representative images are shown at the bottom. Scale bars, 50 μm. *P* values were calculated by paired two-sided Student's t-test. (d) Heat map of normalized enrichment scores (NES) of phosphosite-specific signatures in 31 paired tumor and non-tumor samples. The color in row i and column j indicates that the i-th phosphosite-specific signature is enriched (red; $P < 0.05$), depleted (blue; $P < 0.05$), or non-significant (grey) in the j-th sample. The top boxplot shows the number of enriched, depleted, and non-significant phosphosite-specific signatures for each sample. *P* values (without correction for multiple testing) were calculated by one-sided permutation test. (e) Heat map of NES of phosphosite-specific signatures that are significantly dysregulated between the tumor and non-tumor samples (paired two-sided Student's t-test, BH adjusted $P < 0.01$). (f) Proteomic analysis of genomic altered pathways. Changes in protein expression and phosphorylation of EC mutant genes reported in the literature are shown. Correlation between protein expression levels and patient risk was assessed by the "X-tile" method, and the two-sided log-rank *P* value was calculated by a log-rank test on overall survival difference of two groups divided by the optimal cut-point. The gene interactions were obtained from the KEGG pathway. (g) Comparison of genomic alterations and protein expression changes of genomic altered pathways in EC. Alteration frequencies are expressed as the percentage of cases. SNV/indel is highlighted in dark grey, deletion in blue, and amplification in red. For proteins, fold changes of protein expression levels are shown. No significant difference is highlighted in dark grey, decrease in blue, and increase in red. (h) Proteomic analysis of metastasis and immune response-related genes in Fig. 2g. Changes in protein expression and phosphorylation, and correlation between protein expression levels and patient risk are shown as in (f). The protein-protein interactions were obtained from the STRING database. (i) Comparison of genomic alterations and protein expression changes of metastasis and immune response-related genes in Fig. 2g. Genomic alterations and changes in protein expression are shown as in (g). The protein-protein interactions were obtained from the STRING database.

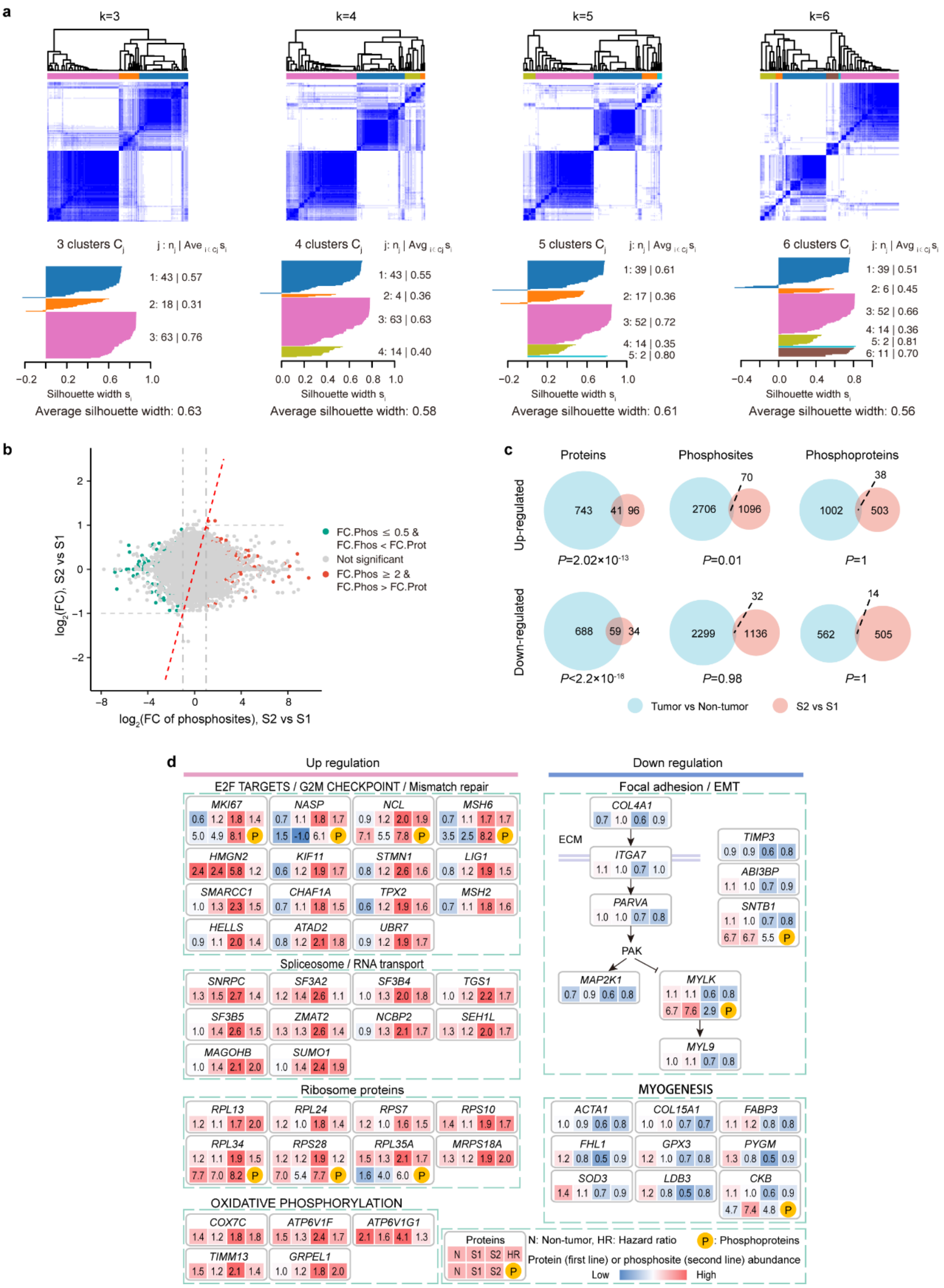
a
k=3
k=4
k=5
k=6
3 clusters C$_j$
j : n$_j$ | Ave$_{i \in Cj}$ s$_i$
1: 43 | 0.57
2: 18 | 0.31
3: 63 | 0.76
Silhouette width s$_i$
Average silhouette width: 0.63
4 clusters C$_j$
j: n$_j$ | Avg$_{i \in Cj}$ s$_i$
1: 43 | 0.55
2: 4 | 0.36
3: 63 | 0.63
4: 14 | 0.40
Average silhouette width: 0.58
5 clusters C$_j$
1: 39 | 0.61
2: 17 | 0.36
3: 52 | 0.72
4: 14 | 0.35
5: 2 | 0.80
Average silhouette width: 0.61
6 clusters C$_j$
1: 39 | 0.51
2: 6 | 0.45
3: 52 | 0.66
4: 14 | 0.36
5: 2 | 0.81
6: 11 | 0.70
Average silhouette width: 0.56
b
log$_2$(FC), S2 vs S1
log$_2$(FC of phosphosites), S2 vs S1
FC.Phos ≤ 0.5 & FC.Phos < FC.Prot
Not significant
FC.Phos ≥ 2 & FC.Phos > FC.Prot
c
Proteins
Phosphosites
Phosphoproteins
Up-regulated
743
41
96
P=2.02×10^{-13}
2706
70
1096
P=0.01
1002
38
503
P=1
Down-regulated
688
59
34
P<2.2×10^{-16}
2299
32
1136
P=0.98
562
14
505
P=1
Tumor vs Non-tumor
S2 vs S1
d
Up regulation
E2F TARGETS / G2M CHECKPOINT / Mismatch repair
MKI67
NASP
NCL
MSH6
HMGN2
KIF11
STMN1
LIG1
SMARCC1
CHAF1A
TPX2
MSH2
HELLS
ATAD2
UBR7
Spliceosome / RNA transport
SNRPC
SF3A2
SF3B4
TGS1
SF3B5
ZMAT2
NCBP2
SEH1L
MAGOHB
SUMO1
Ribosome proteins
RPL13
RPL24
RPS7
RPS10
RPL34
RPS28
RPL35A
MRPS18A
OXIDATIVE PHOSPHORYLATION
COX7C
ATP6V1F
ATP6V1G1
TIMM13
GRPEL1
Down regulation
Focal adhesion / EMT
COL4A1
ECM
ITGA7
PARVA
PAK
MAP2K1
MYLK
MYL9
TIMP3
ABI3BP
SNTB1
MYOGENESIS
ACTA1
COL15A1
FABP3
FHL1
GPX3
PYGM
SOD3
LDB3
CKB
Proteins
N S1 S2 HR
N S1 S2 P
N: Non-tumor, HR: Hazard ratio
P: Phosphoproteins
Protein (first line) or phosphosite (second line) abundance
Low
High

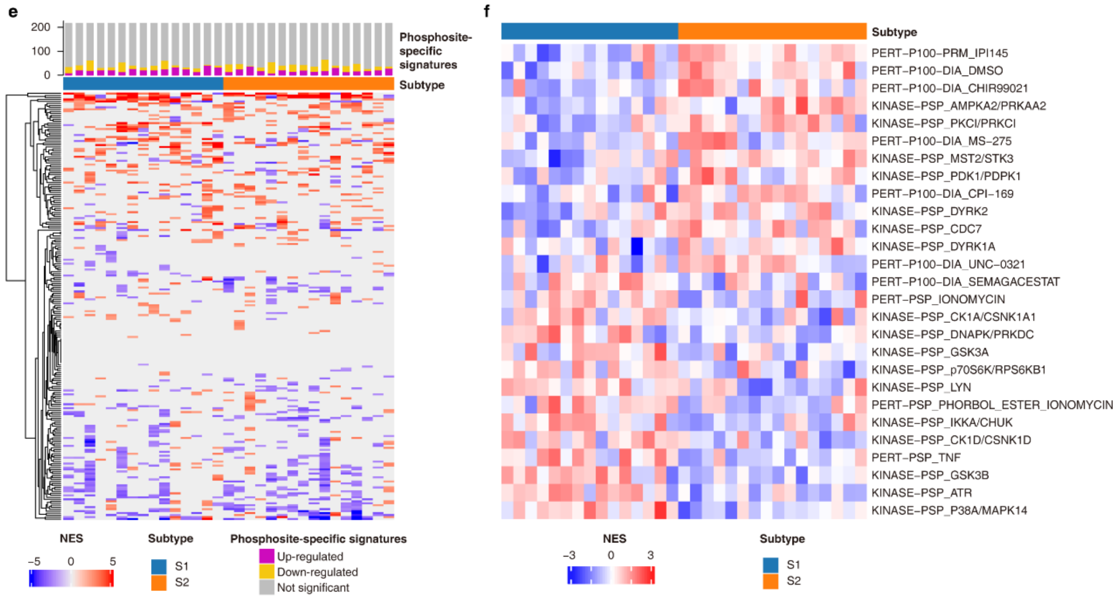

Supplementary Figure 4. Molecular subtypes of EC defined by proteomic analysis.

(a) Consensus clustering of EC tumor samples. The upper panel shows consensus matrices of the 124 EC samples based on clusters from 3 to 6 (k = 3, 4, 5 or 6). Consensus clustering was performed on the top 25% most-variant proteins in Prot5. The bottom panel shows the silhouette-width plots. (b) Comparison of the changes of phosphosite abundance (FC.Phos) with those of the corresponding protein abundance (FC.Prot). The red dashed line indicates the diagonal line. Green colors indicate significantly down-regulated phosphosites (BH adjusted *P* value < 0.01 & FC.Phos ≤ 0.5 & FC.Phos < FC.Prot). Red colors indicate significantly up-regulated phosphosites (BH adjusted *P* value < 0.01 & FC.Phos ≥ 2 & FC.Phos > FC.Prot). Other phosphosites are colored in grey. *P* values were calculated by two-sided Wilcoxon rank-sum test. (c) Comparison of differential proteins, phosphosites, and phosphoproteins between tumor and non-tumor with those between S2 and S1. *P* values were calculated by hypergeometric test. (d) Protein expression ratios (phosphosite abundance) in non-tumor tissues, subtype S1, and S2 and their associations with survival outcome in dysregulated pathways. For phosphoproteins, the abundance of phosphosite with the maximal fold change (FC) is shown. Red indicates high expression and blue indicates low expression. (e) Heat map of normalized enrichment scores (NES) of phosphosite-specific signatures in 15 patients belonged to S1 and 16 belonged to the S2 subtype. Each row represents a phosphosite-specific signature, and each column represents a sample. Phosphosite-specific signature can be enriched (red; *P* < 0.05), depleted (blue; *P* < 0.05), or not significant (grey). The top barplot

shows the number of enriched, depleted, and insignificant phosphosite-specific signatures for each sample. *P* values (without correction for multiple testing) were calculated by one-sided permutation test. (f) Heat map of NES of phosphosite-specific signatures that are significantly dysregulated between the S1 and S2 subtype (unpaired two-sided Student's t-test, $P < 0.05$).

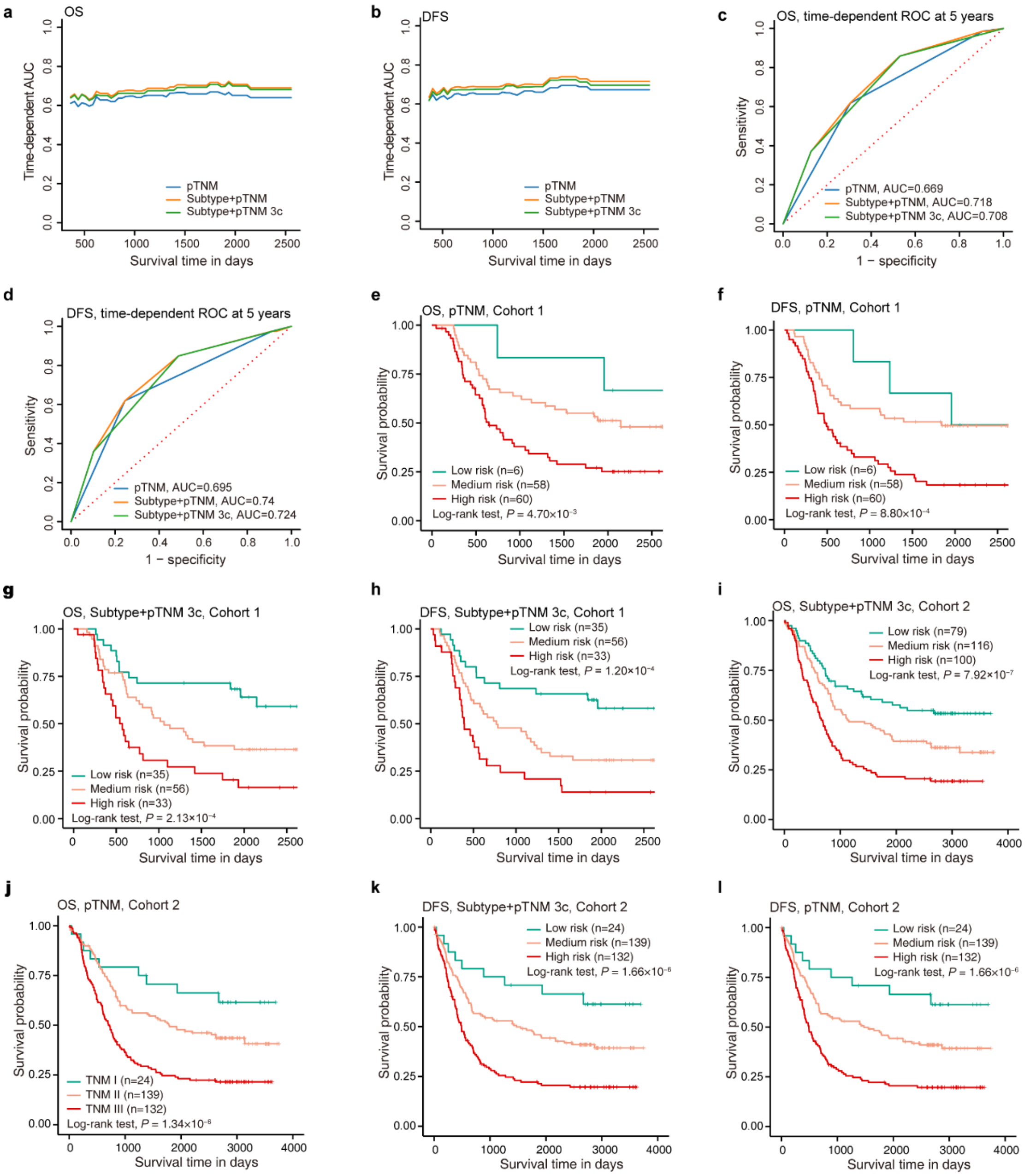

Supplementary Figure 5. Prognostic performance of molecular subtype we defined and that of pTNM stage.

(a, b) Area under the time-dependent ROC curve (TDROC) for OS (a) or DFS (b) of the 124 patients in Cohort 1. Blue, yellow, and green represent the TDROC of pTNM, “Subtype+pTNM”, and “Subtype+pTNM 3c” model, respectively. (c, d) TDROC for OS (C) or DFS (D) at five years. (e, f) Kaplan–Meier curves of OS (e) or DFS (f) for the three groups of patients stratified by pTNM stage in Cohort 1. (g, h) Kaplan–Meier curves of OS (g) or DFS (h) for patients in the three groups stratified by “Subtype+pTNM 3c” model from the 124 patients in Cohort 1. (i, j) Kaplan–Meier curves of OS for patients in the three groups stratified by “Subtype+pTNM 3c”

model (i) or pTNM stage (j) from the independent EC Cohort 2. (k, l) Kaplan–Meier curves of DFS for the three groups of patients stratified by “Subtype+pTNM 3c” model (k) or pTNM stage (l) in Cohort 2. All *P* values in (e-l) were calculated by two-sided log-rank test.

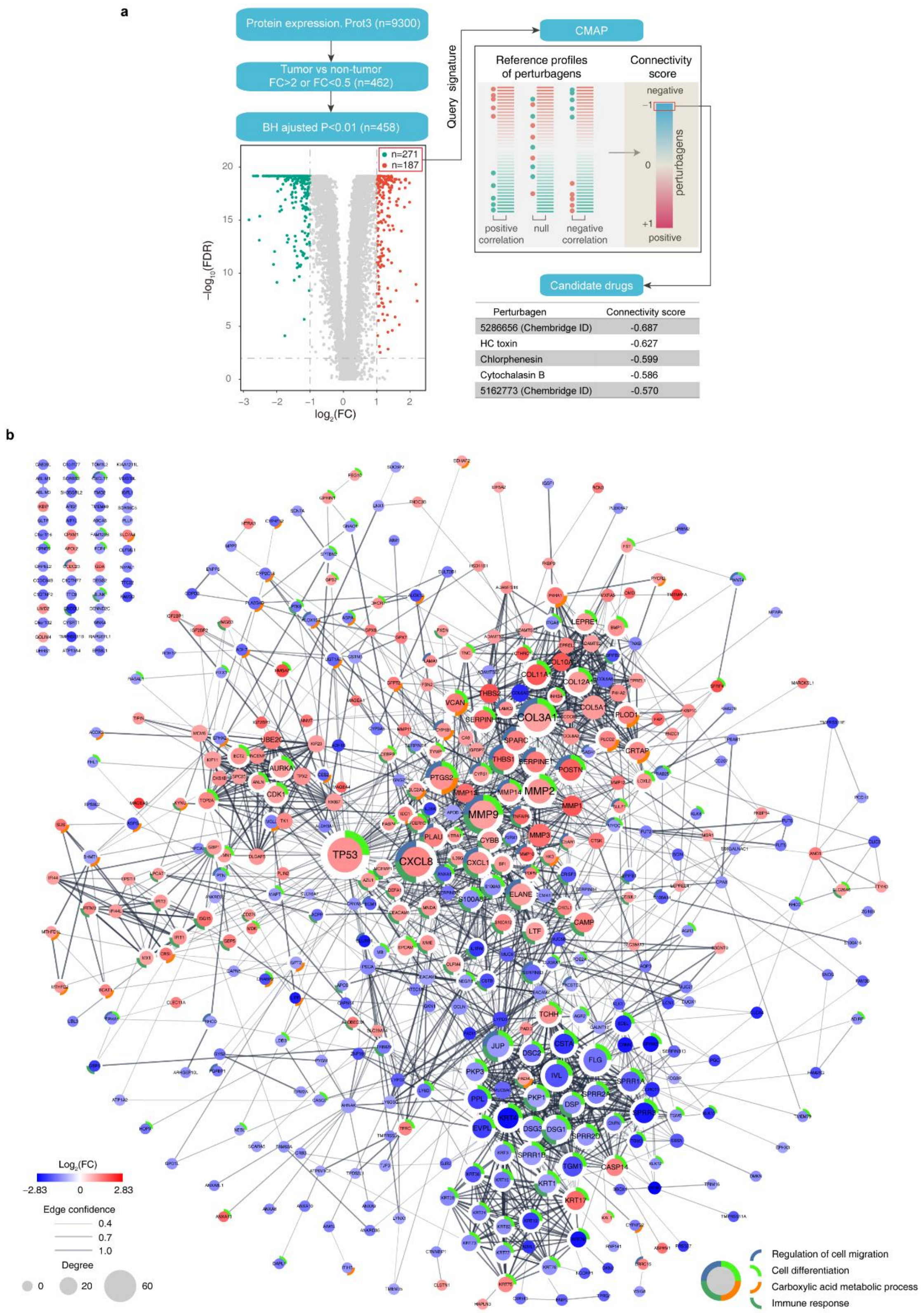

Supplementary Figure 6. CMAP-based drug prediction for EC patients.

(a) Workflow of drug prediction. Volcano plot indicates proteins that are differentially expressed between tumor and non-tumor samples. Red and green represent proteins with fold change larger than 2 and BH adjusted P < 0.01. Other genes are colored in grey. P values were

calculated by two-sided Wilcoxon rank-sum test. The 187 upregulated and 271 downregulated proteins were used as the query signature to match the reference profiles of perturbagens in CMAP to calculate connectivity scores. Perturbagens were sorted by connectivity score in increasing order, and the top perturbagens were predicted as candidate drugs. (b) Protein-protein interaction network of the query signature in (a). The protein-protein interactions were obtained from the STRING database. The width of the line indicates the edge confidence. Upregulated proteins are colored in red, and downregulated proteins in blue. Node size represents the degree of the protein. Proteins involved in regulation of cell migration, cell differentiation, carboxylic acid metabolic process, and immune response are marked with blue, green, orange, and light green, respectively.

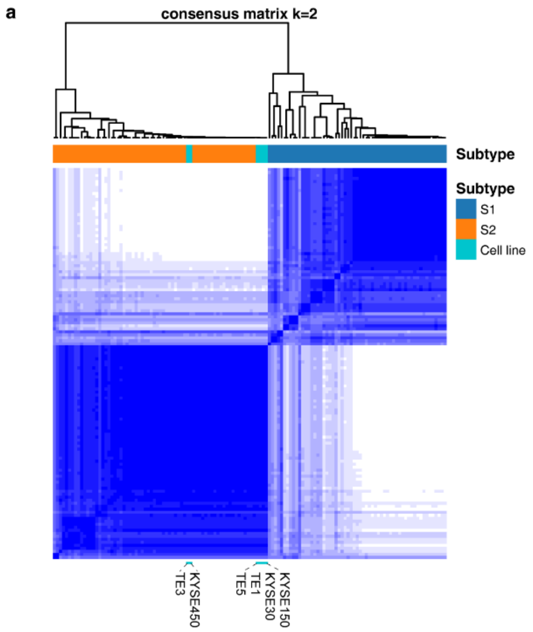

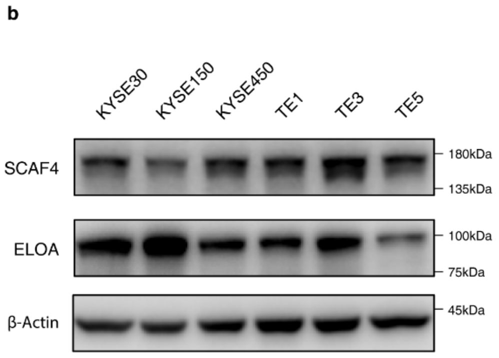

Supplementary Figure 7. Consensus clustering and immunoblotting analysis of subtype signature for EC cell lines.

(a) Consensus clustering of 124 EC tumor samples and six EC cell lines including KYSE30, KYSE150, KYSE450, TE1, TE3, and TE5. (b) Western blotting analysis of ELOA and SCAF4 in the six EC cell lines as described in (a). Each experiment was repeated three times independently with similar results.

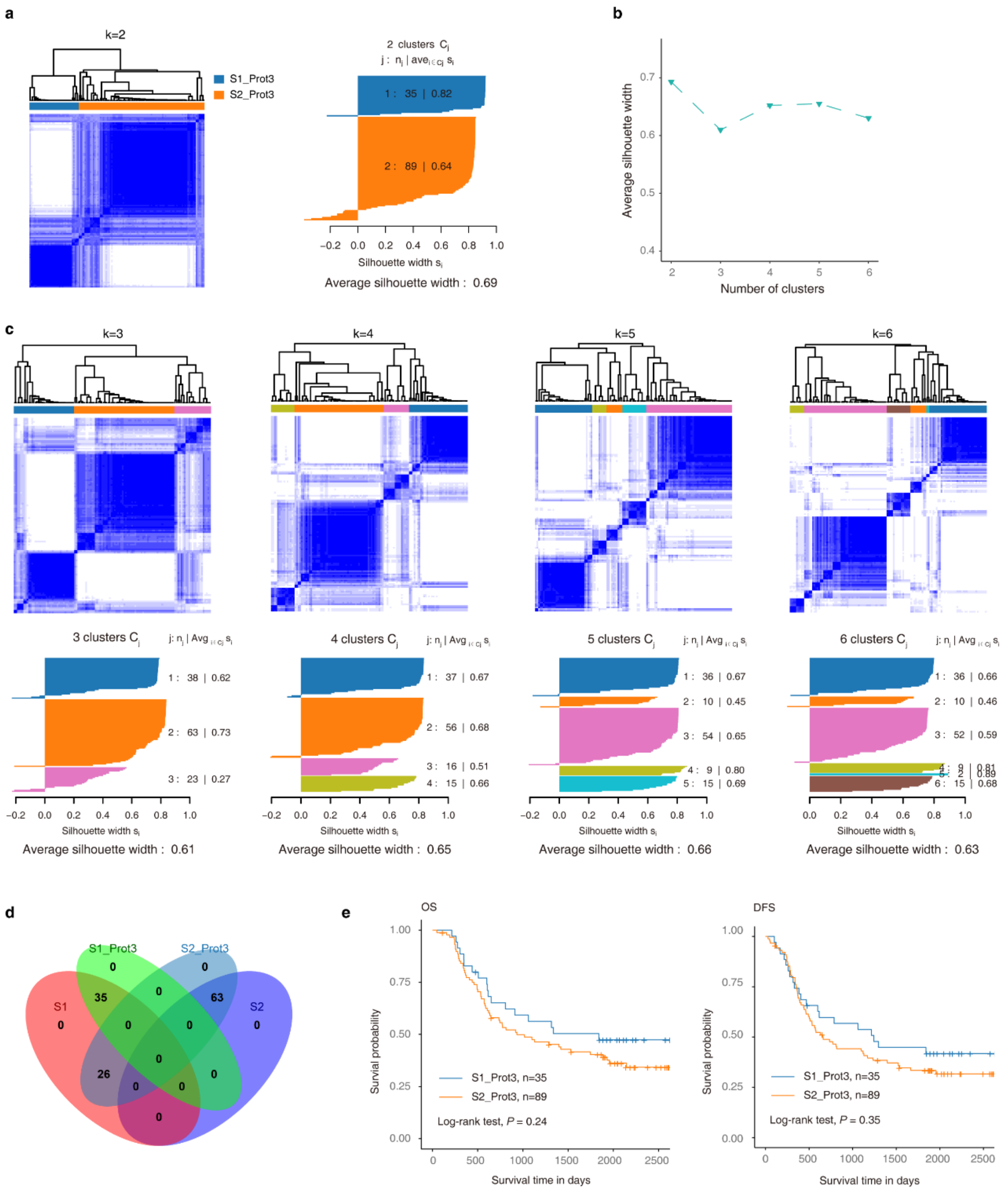

Supplementary Figure 8. Molecular subtypes of EC defined by consensus clustering using the proteins in Prot3 shown in Supplementary Figure 2a.

(a) Consensus clustering of EC tumor samples. The left panel shows consensus matrices of the 124 EC samples with two clusters (k = 2). Consensus clustering was performed on the top 25% of the most variable proteins in Prot3 as described in Supplementary Figure 2a. The right panel shows the silhouette-width plot. (b) Average silhouette-width plot. The average silhouette width takes the maximum value when number of clusters was 2 (k = 2). (c) Consensus matrices of the 124 EC samples based on clusters from 3 to 6 (k = 3, 4, 5 or 6). The bottom panel shows

the silhouette-width plots. (d) Venn diagram of subtype S1, S2, S1_Prot3, and S2_Prot3. (e) Kaplan–Meier curves of overall survival (left) and disease-free survival (right) for subtype S1_Prot3 and S2_Prot3. *P* values are calculated by two-sided log-rank test.
