# Supplementary material for: Large-scale and high-resolution mass spectrometry-based proteomics profiling defines molecular subtypes of esophageal cancer for therapeutic targeting
Source: Nat Commun. 2021 Aug 16;12:4961. doi: 10.1038/s41467-021-25202-5 (PMC8368010; doi:10.1038/s41467-021-25202-5)
Supplement: Supplementary file 2 — Description of Additional Supplementary Files [file 41467_2021_25202_MOESM2_ESM.pdf]

Description of Additional Supplementary Files

File Name: Supplementary Data 1
Description: **Clinical information and proteomic data of the 124 patients with esophageal cancer (EC).** (**A**) Clinical data. (**B**) Expression ratios (tumor versus non-tumor) of 9,300 proteins quantified with high confidence in at least half samples. (**C**) Abundance of 61,471 phosphosites quantified in at least half samples. (**D**) Subcellular distribution of proteins and phosphoproteins. *P* values were calculated by two-sided Wilcoxon signed-rank test.

File Name: Supplementary Data 2
Description: **Dysregulated proteins and pathways in EC.** (**A**) Differentially expressed proteins (tumor versus non-tumor). (**B**) Esophageal specific proteins. *P* values were calculated by two-sided Wilcoxon signed-rank test. (**C**) Differentially expressed phosphosites (tumor versus non-tumor). (**D, E**) Enrichment analysis of differentially expressed proteins (D) and phosphorylated proteins (E) (tumor versus non-tumor). *P* values were calculated by hypergeometric test. (**F**) Pathways used for heatmap. (**G**) EC-associated risk proteins. *P* values were calculated by two-sided Wilcoxon signed-rank test.

File Name: Supplementary Data 3
Description: **Proteomic analysis of the mutated genes.** (**A**) Survival analysis for significantly mutated genes. The two-sided Cox *P* values were calculated using the Cox PH model. (**B**) Proteomic analysis of mutated genes in the genomic altered pathways. *P* values were calculated by two-sided Wilcoxon signed-rank test. Log-rank P values were by two-sided log-rank test. (**C**) Phosphorylation sites of mutated genes in the genomic altered pathways. *P* values were calculated by two-sided Wilcoxon signed-rank test.

File Name: Supplementary Data 4
Description: **Molecular subtypes of EC.** (**A**) Molecular subtype. (**B**) Univariate and multivariate analysis of factors associated with overall survival (OS) and disease-free survival (DFS). ^{a}P values were calculated by univariate Cox regression analysis; ^{b}P values were calculated by multivariate Cox regression analysis. (**C**) Clinicopathologic correlation. *P* values were calculated by the χ^2-test or Fisher's exact test, unless otherwise stated. *Two-sided Wilcoxon rank-sum test. (**D, E**) Differentially expressed proteins (D) and phosphosites (E) (S2 versus S1). (**F, G**) Enrichment analysis of differentially expressed proteins (F) and phosphorylated proteins (G) (S2 versus S1). *P* values were calculated by hypergeometric test. Q values were adjusted *P* values by BH correction.

File Name: Supplementary Data 5
Description: **Subtype prediction and immunohistochemical (IHC) validation.** (**A**) Frequencies of identified signatures. (**B**) Average predictive performance of the 11 signatures. (**C**) Detailed clinical information of the 295 EC patients that used for immunohistochemical analysis. (**D**) Immunohistochemistry scores and the predicted subtypes.

File Name: Supplementary Data 6
Description: **Drug prediction and validation.** (**A**) Query signature for EC. (**B**) Drug prediction for EC. *P* values were calculated by one-sided permutation test. (**C**) Query signature for S2-subtype. (**D**) Drug prediction for S2-subtype. *P* values were calculated by one-sided permutation test. (**E**) IC 50 values for Sulconazole, Menadlone, and GW8510 in EC cell lines as indicated. (**F**) Proteomic data of KYSE150 cells. (**G-I**) Expression changes of proteins differentially expressed between S1 and S2 subtype in response to GW8510 (**G**), Menadione (**H**) and Sulconazole (**I**) in KYSE150 cells. The two-sided Cox *P* values were calculated using the Cox PH model.
