# Supplementary material for: Large-scale and high-resolution mass spectrometry-based proteomics profiling defines molecular subtypes of esophageal cancer for therapeutic targeting
Source: Nat Commun. 2021 Aug 16;12:4961. doi: 10.1038/s41467-021-25202-5 (PMC8368010; doi:10.1038/s41467-021-25202-5)
Supplement: Supplementary file 9 — Reporting Summary [file 41467_2021_25202_MOESM9_ESM.pdf]

nature research

Corresponding author(s): En-Min Li, Wen Liu, Li-Yan Xu

Last updated by author(s): Jul 1, 2021

Reporting Summary

Nature Research wishes to improve the reproducibility of the work that we publish. This form provides structure for consistency and transparency in reporting. For further information on Nature Research policies, see our Editorial Policies and the Editorial Policy Checklist.

Statistics

For all statistical analyses, confirm that the following items are present in the figure legend, table legend, main text, or Methods section.

n/a	Confirmed	
☐	☒	The exact sample size (*n*) for each experimental group/condition, given as a discrete number and unit of measurement
☐	☒	A statement on whether measurements were taken from distinct samples or whether the same sample was measured repeatedly
☐	☒	The statistical test(s) used AND whether they are one- or two-sided<br>*Only common tests should be described solely by name; describe more complex techniques in the Methods section.*
☐	☒	A description of all covariates tested
☐	☒	A description of any assumptions or corrections, such as tests of normality and adjustment for multiple comparisons
☐	☒	A full description of the statistical parameters including central tendency (e.g. means) or other basic estimates (e.g. regression coefficient) AND variation (e.g. standard deviation) or associated estimates of uncertainty (e.g. confidence intervals)
☐	☒	For null hypothesis testing, the test statistic (e.g. *F*, *t*, *r*) with confidence intervals, effect sizes, degrees of freedom and *P* value noted<br>*Give P values as exact values whenever suitable.*
☒	☐	For Bayesian analysis, information on the choice of priors and Markov chain Monte Carlo settings
☒	☐	For hierarchical and complex designs, identification of the appropriate level for tests and full reporting of outcomes
☐	☒	Estimates of effect sizes (e.g. Cohen's *d*, Pearson's *r*), indicating how they were calculated

Our web collection on statistics for biologists contains articles on many of the points above.

Software and code

Policy information about availability of computer code

Data collection	Xcalibur (version 3.0), Vectra (version 2.0.8), Nuance (version 3.0), inform (version 1.2)
Data analysis	R (version 3.6.2), Proteome Discoverer (version 2.2), GraphPad Prism 7 (version 7.0.4), ggbiplot (version 0.55), pheatmap (version 1.0.12), limma (version 3.42.2), ConsensusClusterPlus (version 1.50.0), survival (version 3.1-8), survcomp (version 1.36.1), survivalROC (version 1.0.3), survminer (version 0.4.6), mlr (version 2.17.0), Metascape (version 3.5), PTM-SEA/ssGSEA (version 2.0)

For manuscripts utilizing custom algorithms or software that are central to the research but not yet described in published literature, software must be made available to editors and reviewers. We strongly encourage code deposition in a community repository (e.g. GitHub). See the Nature Research guidelines for submitting code & software for further information.

Data

Policy information about availability of data

All manuscripts must include a data availability statement. This statement should provide the following information, where applicable:

- Accession codes, unique identifiers, or web links for publicly available datasets
- A list of figures that have associated raw data
- A description of any restrictions on data availability

The data that support the findings of this study—including clinical information, and proteome and phosphoproteome data—are available within the paper and its Supplementary Information. The raw files of proteome and phosphoproteome datasets can be obtained from PRIDE database (https://www.ebi.ac.uk/pride/, accession number PXD021701) or iProX database (www.iprox.org, accession number IPX0002501000). PTM signatures database (PTMsigDB, v1.9.0) was downloaded from http://prot-shiny-vm.broadinstitute.org:3838/ptmsigdb-app/. Source data are provided with this paper.

Field-specific reporting

Please select the one below that is the best fit for your research. If you are not sure, read the appropriate sections before making your selection.

☒ Life sciences ☐ Behavioural & social sciences ☐ Ecological, evolutionary & environmental sciences

For a reference copy of the document with all sections, see nature.com/documents/nr-reporting-summary-flat.pdf

Life sciences study design

All studies must disclose on these points even when the disclosure is negative.

Sample size	No statistical method was used to predetermine sample size. The biological experiments were performed with at least three biological replicates to allow statistical significance testing through student t-test. The sample size of EC patients was based on published papers in the proteomic field (PMID: 30814741; 31585088; 30645970).
Data exclusions	No data were excluded.
Replication	Information provided in Figure legends.
Randomization	For the TMT-11plex experiments, samples of EC patients were randomly divided into 25 groups for protein quantification. For Xenograft assay, mice were randomly assigned to experimental or control groups.
Blinding	The investigator who measured protein expression by mass spectrometry was blinded to patient information in the 25 groups.<br>The investigators who performed IHC were blinded to clinical information of 295 EC patients.

Reporting for specific materials, systems and methods

We require information from authors about some types of materials, experimental systems and methods used in many studies. Here, indicate whether each material, system or method listed is relevant to your study. If you are not sure if a list item applies to your research, read the appropriate section before selecting a response.

Materials & experimental systems

n/a	Involved in the study	
☐	☒	Antibodies
☐	☒	Eukaryotic cell lines
☒	☐	Palaeontology and archaeology
☐	☒	Animals and other organisms
☐	☒	Human research participants
☒	☐	Clinical data
☒	☐	Dual use research of concern

Methods

n/a	Involved in the study	
☒	☐	ChIP-seq
☒	☐	Flow cytometry
☒	☐	MRI-based neuroimaging

Antibodies

Antibodies used	Rabbit anti-ELOA polyclonal antibody (NBP1-87040, Novus) ,<br>Rabbit anti-SCAF4 polyclonal antibody (PA5-36611, Thermo fisher),<br>Rabbit anti-MX1 polyclonal antibody (13750-1-AP, Proteintech),<br>Rabbit anti-OAS3 polyclonal antibody (21915-1-AP, Proteintech),<br>Mouse anti-IFIT1 monoclonal antibody (TA500948S, Origene)<br>Mouse Beta Actin Monoclonal Antibody (Catalog number: 66009-1-Ig, Proteintech)
Validation	NBP1-87040, validated for immunohistochemistry by manufacturer: https://www.novusbio.com/products/elongin-a-antibody_nbp1-87040#datasheet<br>PA5-36611, validated for immunohistochemistry by manufacturer: https://www.thermofisher.com/order/genome-database/dataSheetPdf?producttype=antibody&productsubtype=antibody_primary&productId=PA5-36611&version=130<br>13750-1-AP, validated for western blotting by manufacturer: https://www.ptglab.com/Products/MX1-Antibody-13750-1-AP.htm<br>21915-1-AP, validated for western blotting by manufacturer: https://www.ptglab.com/Products/OAS3-Antibody-21915-1-AP.htm<br>TA500948S, validated for western blotting by manufacturer: https://www.thermofisher.cn/cn/zh/antibody/product/IFIT1-Antibody-OTI3G8-TrueMAB-Mouse-Monoclonal/TA500948S?adobe_mc=MCMID%7C73652251332455499110453698239322138026%7CMCAID%3D30470E9946E99DCC-400012C5A497DFB2%7CMCORGID%3D5B135A0C5370E6B40A490D44@AdobeOrg%7CTS%3D1614293705<br>Beta-Actin (66009-1-Ig), validated for western blotting by manufacturer: https://www.ptgcn.com/products/Pan-Actin-Antibody-66009-1-Ig.htm

Eukaryotic cell lines

Policy information about cell lines

Cell line source(s)	ESCC cell lines (KYSE30, KYSE150, KYSE450, TE1, TE3 and TE5) were purchased from Cell Bank of Type Culture Collection of Chinese Academy of Sciences.
Authentication	All of the cell lines used are not found in the cell lines registered as misidentified cell lines in the International Cell Line Authentication Committee (ICLAC).
Mycoplasma contamination	All cell lines were tested negative for mycoplasma contamination by PCR.
Commonly misidentified lines (See ICLAC register)	No such misidentified lines were used in the study.

Animals and other organisms

Policy information about studies involving animals; ARRIVE guidelines recommended for reporting animal research

Laboratory animals	Animal experiments were carried out according to the program approved by the Medical Animal Care and Welfare Committee of Shantou University Medical College. Nude mice (Vital River Laboratories Animal Technology, Beijing, China) aged 3-5 weeks were randomly divided into four groups. The tumor was resected and weighed after the mice were euthanized with excessive $CO2$ 30 days after inoculation. The feeding conditions were specific pathogen free animal laboratory with 28 °C and 50% humidity, providing sufficient water and diet.
Wild animals	No wild animals were used in the study.
Field-collected samples	No field-collected samples were used in the study.
Ethics oversight	Animal experiments were carried out according to the program approved by Animal Research Committee of Shantou administrative center.

Note that full information on the approval of the study protocol must also be provided in the manuscript.

Human research participants

Policy information about studies involving human research participants

Population characteristics	The 124 EC samples in Cohort 1 included 27 females and 97 males at the mean age of 58.5 (the age range from 41 to 77) (detailed clinical data were provided in supplementary data 1a).<br>The 295 EC samples in Cohort 2 included 66 females and 229 males at the mean age of 58.9 (the age range from 39 to 88) (detailed clinical data were provided in supplementary data 5c).<br>Only resected samples from surgical patients with written informed consent were included.
Recruitment	The 124 EC samples in Cohort 1 were obtained from the Shantou Central Hospital. All the patients underwent curative resection from June 2011 to December 2013.<br>The second independent cohort (Cohort 2) included 295 EC patients that underwent curative resection from November 2007 to January 2011 at the Shantou Central Hospital.<br>There was no selection bias.
Ethics oversight	Ethical approval was obtained from the ethical committee of the Central Hospital of Shantou City and the ethical committee of the Shantou University Medical College.

Note that full information on the approval of the study protocol must also be provided in the manuscript.
